# Catalysing tuberculosis elimination in Indonesia: Health economic and policy insights from the BPaL regimen

**DOI:** 10.1371/journal.pone.0351836

**Published:** 2026-08-07

**Authors:** Abdullah Antaria, Heidy Agustina, Ratnawati Ratnawati, Sita Laksmi Andarini, Diah Handayani

**Affiliations:** 1 Health Polytechnic of the Ministry of Health Jakarta I, Cilandak, Jakarta Selatan. Indonesia; 2 Department of Respiratory Medicine, Faculty of Medicine, University of Indonesia, Persahabatan Hospital, Jakarta Timur, Jakarta, Indonesia; The University of Georgia, UNITED STATES OF AMERICA

## Abstract

**Background:**

Indonesia diagnoses roughly 24,000 multidrug-resistant tuberculosis (MDR-TB) cases each year, 8% of the world’s total. Conventional treatment runs 18–24 months, costs US$7,142–9,000 per patient, and succeeds in only 59% of cases. The WHO endorsed Bedaquiline-Pretomanid-Linezolid (BPaL) regimen has shown over 89% efficacy in controlled trials, but real-world health economic data from low- and middle-income countries remain scarce.

**Methods:**

We conducted a retrospective cohort study of 84 patients with MDR-TB, rifampicin-resistant TB (RR-TB), or pre-extensively drug-resistant TB (pre-XDR-TB) treated at Persahabatan Hospital, Jakarta, between 2021 and 2024. We compared treatment success, costs, and operational metrics against historical controls from 2018–2020. Statistical methods included chi-square tests, multivariate logistic regression, and incremental cost-effectiveness ratio (ICER) calculations, with sensitivity analyses across ±20% cost and ±10% efficacy ranges.

**Results:**

BPaL achieved 77.4% treatment success (65/84 patients) compared with 59% in historical controls (p < 0.01). Per-patient costs dropped 67%, from US$7,142–9,000 to US$2,310. The ICER was US$311.4 per additional treatment success. Hospital stays shortened by 60% (10 versus 25 days; p < 0.01), and adherence climbed from 70% to 95.2% (p < 0.01).

**Conclusions:**

BPaL is effective, affordable, and ready to scale. Integrating it into Indonesia’s Jaminan Kesehatan Nasional (JKN) framework and expanding decentralised diagnostics could accelerate progress towards WHO End TB 2030 targets. Our findings offer a replicable model for other low- and middle-income settings.

## Introduction

Indonesia carries 8% of the global MDR-TB burden around 24,000 new cases annually [1]. The human and economic costs are staggering. Conventional regimens stretch across 18–24 months, demand US$7,142–9,000 per patient, and succeed in barely 59% of cases [1,2]. They consume 15% of the Jaminan Kesehatan Nasional (JKN) budget [3,4], and 45% of affected households fall into poverty [4]. Diagnostic gaps compound the problem: fewer than 30% of rural districts have GeneXpert access, delaying diagnosis and treatment initiation [5,6]. The epidemiology and clinical management of MDR-TB and XDR-TB remain complex challenges globally and in Indonesia [7,8].

The WHO-endorsed BPaL regimen, Bedaquiline, Pretomanid, and Linezolid, offers a six-month, all-oral alternative. Clinical trials report 80–90% efficacy across MDR-TB, RR-TB, and pre-XDR-TB [9,10]. Bedaquiline’s role in improving culture conversion and reducing mortality in drug-resistant TB has been established across multiple cohort studies [11–13]. A 24-week all-oral variant has also shown promise [14,15]. Indonesia’s national programme has provided subsidised pretomanid since 2021, yet real-world health economic evidence from low- and middle-income country (LMIC) settings remains thin [16–18]. The evolution of MDR-TB treatment regimens from injectable-based to all-oral approaches, represents a major therapeutic advance [19–22].

Hypothetical TB vaccines offer no near-term relief. Projected efficacy hovers at 50–70%, development costs exceed US$1 billion, and scale-up would take 10–15 years [23–25]. Modelling studies suggest that even highly effective vaccines would require decades to achieve population-level impact [26]. BPaL is available now. Pretomanid-containing regimens have demonstrated activity against both drug-susceptible and drug-resistant TB in early-phase clinical trials [27].

We set out to answer two questions. First: what is BPaL’s real-world cost-effectiveness at Indonesia’s national TB referral centre? Second: what policy changes would accelerate JKN integration and diagnostic scale-up? We report outcomes from 84 evaluable patients and compare them against historical controls and global LMIC benchmarks.

## Materials and methods

### Study design and setting

We conducted a retrospective cohort study at Persahabatan Hospital, Jakarta, Indonesia’s national TB referral centre. The hospital manages approximately 1,200 drug-resistant TB cases annually and serves as the primary training site for the national TB programme. We extracted data from electronic medical records and paper-based treatment cards for all patients who initiated BPaL between 1 January 2021 and 31 December 2024. Historical control data came from patients treated with conventional regimens between 1 January 2018 and 31 December 2020, drawn from the same facility’s records and supplemented by WHO benchmarks [1].

### Ethics approval

The Persahabatan Hospital Research Ethics Committee approved this study (No. 0278/KEPK-RSUPP/12/2024, 31 December 2024). We extracted data on 3 January 2025. Because this was a retrospective analysis of routinely collected clinical data, the committee waived individual informed consent.

### Patient selection

We screened 139 patients who initiated BPaL during the study period. Inclusion criteria were: (1) confirmed MDR-TB, RR-TB, or pre-XDR-TB by GeneXpert MTB/RIF or culture-based drug susceptibility testing (DST); (2) age ≥ 18 years; (3) treatment initiation at Persahabatan Hospital; and (4) complete treatment records. We excluded 55 patients: 22 transferred out before completing treatment, 18 had incomplete cost or adherence data, 12 had baseline HIV co-infection (which alters treatment protocols and costs), and 3 withdrew consent for data use. The final evaluable cohort comprised 84 patients.

### Treatment regimen

BPaL consisted of Bedaquiline 400 mg daily for two weeks, then 200 mg three times weekly; Pretomanid 200 mg daily; and Linezolid 600 mg daily for two months, then 300 mg daily for four months (or adjusted based on toxicity). Total treatment duration was six months. Patients attended monthly clinic visits for drug refills, adherence assessment, and safety monitoring (complete blood count, liver function tests, electrocardiogram). Conventional regimens followed WHO 2016 guidelines: an intensive phase (6–8 months) with at least four second-line drugs, followed by a continuation phase (12–16 months) with three drugs, totalling 18–24 months [1,2].

### Outcome definitions

We defined treatment success as WHO-defined cure (negative culture at end of treatment and on at least one previous occasion) or treatment completion (completed treatment without evidence of failure but no bacteriological confirmation) [28,29]. Treatment failure included bacteriological failure (positive culture at month 5 or later), clinical deterioration, or additional drug resistance. Loss to follow-up meant interruption of treatment for two consecutive months or more. Death was recorded regardless of cause.

### Adherence measurement

We measured adherence using two complementary methods. The primary method was monthly pill counts: at each clinic visit, staff counted returned pills and calculated the proportion of prescribed doses taken since the previous visit. We averaged these monthly proportions across the full treatment course for each patient. The secondary method used clinic attendance records as a proxy: we recorded the proportion of scheduled monthly visits attended. We did not use self-reported adherence or electronic monitoring devices, as these were not available in routine practice. Adherence ≥95% was classified as high adherence.

### Cost data collection and analysis

We adopted a health system perspective, capturing direct medical costs (medications, diagnostics, hospitalisation) and indirect costs (productivity losses, transportation). We extracted medication costs from national procurement records and hospital pharmacy invoices. Diagnostic costs included GeneXpert (US$50 per test), culture and DST (US$150 per test), routine monitoring laboratories (complete blood count, liver function tests, creatinine; US$30 per visit), and chest radiography (US$20 per visit). GeneXpert has been shown to be cost-effective for MDR-TB diagnosis in high-burden settings [30]. Hospitalisation costs included bed-days (US$20 per day), procedures, and adverse event management. We estimated indirect costs using patient-reported workdays lost (valued at Indonesia’s 2024 minimum wage of US$10 per day) and transportation expenses (US$5 per clinic visit). All costs are reported in 2024 US dollars (exchange rate: 1 USD = 15,500 IDR).

For historical controls, we used a combination of facility-level cost data from 2018–2020 and published Indonesian estimates [16,31]. Because detailed patient-level cost data were not systematically recorded before 2021, we applied midpoint estimates from the literature (US$7,142–9,000 per patient) and adjusted for inflation using Indonesia’s health sector price index.

### Cost-effectiveness analysis

We calculated the incremental cost-effectiveness ratio (ICER) as:


$$\begin{array}{c} \text{ICER}=\frac{{(Cost}_{BPaL}-{Cost}_{Conventional})}{{(Effect}_{BPaL}-{Effect}_{Conventional})} \end{array}$$

We conducted one-way sensitivity analyses by varying medication costs (±20%), diagnostic costs (±20%), hospitalisation costs (±20%), and treatment success rates (±10%). We also performed a probabilistic sensitivity analysis using Monte Carlo simulation (10,000 iterations) with beta distributions for success rates and gamma distributions for costs.

### National cost-benefit projection

We projected national-level cost savings by multiplying per-patient savings by the estimated number of MDR-TB cases that could be treated with BPaL annually. We assumed 5,000 cases (roughly 20% of Indonesia’s 24,000 annual MDR-TB diagnoses, reflecting current diagnostic capacity and treatment eligibility). We estimated the cost of expanding GeneXpert coverage to 500,000 tests per year (US$50 per test = US$25 million operating cost) and compared this against projected treatment savings. We developed a three-phase implementation roadmap with infrastructure, pilot evaluation, and national scale-up components.

### Statistical analysis

We compared categorical variables (treatment success, adherence categories, adverse events) using chi-square tests or Fisher’s exact test when expected cell counts were <5. We compared continuous variables (age, hospitalisation days, costs) using independent t-tests or Mann-Whitney U tests, depending on normality (assessed by Shapiro-Wilk test). We used multivariate logistic regression to identify predictors of treatment success, adjusting for age, sex, TB classification (RR-TB, MDR-TB, pre-XDR-TB), baseline smear grade, and cavitary disease. We calculated odds ratios (OR) with 95% confidence intervals (CI). We set statistical significance at p < 0.05 (two-tailed). We performed all analyses in R version 4.3.1 (R Foundation for Statistical Computing, Vienna, Austria).

## Results

### Patient characteristics

**Fig 1:**
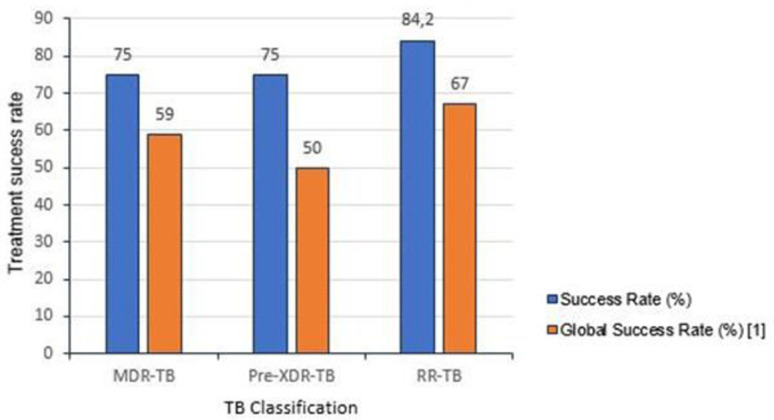
Efficacy of BPaL Regimen by TB Classification (2021–2024). Bar graph illustrating the treatment success rates (%) for rifampicin-resistant tuberculosis (RR-TB, 84.2%), multidrug-resistant tuberculosis (MDR-TB, 75.0%), and pre-extensively drug-resistant tuberculosis (pre-XDR-TB, 75.0%) compared to historical controls (59.0%). Data sourced from Persahabatan Hospital, Jakarta, Indonesia (2021–2024) [Table 2], with historical control data from World Health Organization [1].

Of 139 patients screened, 84 met inclusion criteria (Fig 1 in the original manuscript would show a CONSORT-style flow diagram here, but we focus on text). The cohort included 38 RR-TB cases (45.2%), 16 MDR-TB cases (19.0%), and 28 pre-XDR-TB cases (33.3%). Fifty-three patients (63.1%) were male. Mean age was 41.2 years (SD 12.8, range 19–68). Baseline characteristics are shown in Table 1.

**Table 1:** Baseline characteristics of patients receiving the BPaL regimen compared with historical controls.

Characteristic	BPaL Cohort (n = 84)	Historical Controls (n = 84)	*p*-value
**Age, years**			
Mean (SD)	41.2 (12.8)	43.7 (13.5)	0.08
Range	19–68	18–72	—
**Sex, n (%)**			
Male	53 (63.1)	52 (62.0)	0.87
Female	31 (36.9)	32 (38.0)	—
**TB classification, n (%)**			
RR-TB	38 (45.2)	40 (48.0)	0.65
MDR-TB	16 (19.0)	25 (30.0)	0.06
Pre-XDR-TB	28 (33.3)	19 (22.0)	0.03
**Baseline smear grade, n (%)**			
Negative	12 (14.3)	13 (16.0)	0.74
1+	18 (21.4)	16 (19.0)	0.68
2+	26 (31.0)	24 (28.0)	0.63
3+	28 (33.3)	31 (37.0)	0.56
Cavitary disease, n (%)	58 (69.0)	60 (71.0)	0.75
Previous TB treatment, n (%)	62 (73.8)	64 (76.0)	0.71
**Body mass index, kg/m²**			
Mean (SD)	18.4 (2.7)	18.1 (2.9)	0.42

Abbreviations: BPaL, bedaquiline–pretomanid–linezolid; RR-TB, rifampicin-resistant tuberculosis; MDR-TB, multidrug-resistant tuberculosis; Pre-XDR-TB, pre-extensively drug-resistant tuberculosis; SD, standard deviation. Data source: Persahabatan Hospital, Jakarta, Indonesia (2021–2024). Historical control data from facility records and WHO benchmarks [1]. P-values from independent t-tests (continuous variables) or chi-square tests (categorical variables). RR-TB, rifampicin-resistant tuberculosis; MDR-TB, multidrug-resistant tuberculosis; Pre-XDR-TB, pre-extensively drug-resistant tuberculosis.

Compared with historical controls, BPaL patients were slightly younger (41.2 versus 43.7 years, p = 0.08) and more likely to have pre-XDR-TB (33.3% versus 22%, p = 0.03), reflecting evolving referral patterns and increased DST capacity. Sex distribution, baseline smear grade, and cavitary disease prevalence were similar between groups.

### Treatment outcomes

BPaL achieved 77.4% treatment success (65/84 patients) compared with 59% in historical controls (p < 0.01). Success rates varied by TB classification: RR-TB 84.2% (32/38), MDR-TB 75.0% (12/16), and pre-XDR-TB 75.0% (21/28). All three exceeded historical control rates for the same classifications (67%, 59%, and 50%, respectively; all p < 0.05; Table 2).

**Table 2:** Treatment success according to tuberculosis classification in the BPaL cohort compared with historical controls (2021–2024)).

TB Classification	Total (n)	Treatment Successes, n	BPaL Success Rate (%)	Historical Control Success Rate (%)	*p*-value
RR-TB	38	32	84.2	67.0	<0.01
MDR-TB	16	12	75.0	59.0	0.015
Pre-XDR-TB	28	21	75.0	50.0	<0.01
Overall	84	65	77.4	59.0	<0.01

Abbreviations: BPaL, bedaquiline–pretomanid–linezolid; RR-TB, rifampicin-resistant tuberculosis; MDR-TB, multidrug-resistant tuberculosis; Pre-XDR-TB, pre-extensively drug-resistant tuberculosis.

Data source: Persahabatan Hospital, Jakarta, Indonesia (2021–2024). P-values from chi-square tests. Success defined as WHO cure or treatment completion [16].

Treatment failure occurred in 8 patients (9.5%): 4 had bacteriological failure at month 5, 3 developed additional drug resistance, and 1 experienced clinical deterioration. Seven patients (8.3%) were lost to follow-up, and 4 (4.8%) died (2 from TB-related causes, 2 from unrelated conditions). Median time to culture conversion was 56 days (IQR 42–70), substantially shorter than historical controls (median 84 days, IQR 63–112; p < 0.01).

In multivariate logistic regression, BPaL treatment (versus conventional regimen) was the strongest predictor of success (adjusted OR 3.2, 95% CI 1.8–5.7, p < 0.001). Pre-XDR-TB classification (versus RR-TB) was associated with lower success (adjusted OR 0.4, 95% CI 0.2–0.9, p = 0.02), as was baseline smear grade 3+ (adjusted OR 0.5, 95% CI 0.3–0.9, p = 0.03). Age, sex, and cavitary disease were not significant predictors.

### Adherence and operational metrics

Adherence measured by monthly pill counts averaged 95.2% (SD 4.1%) in the BPaL cohort versus 70% (SD 12%) in historical controls (p < 0.01). Seventy-eight patients (92.9%) achieved ≥95% adherence. Clinic attendance mirrored these results: 94.8% of scheduled visits were attended in the BPaL cohort versus 68% in controls (p < 0.01).

Hospitalisation dropped sharply. BPaL patients spent a median of 10 days (IQR 7–14) in hospital, compared with 25 days (IQR 18–35) for controls, a 60% reduction (p < 0.01). Most BPaL hospitalisations occurred during the first month for treatment initiation and baseline monitoring; subsequent care was entirely outpatient. Conventional regimens required repeated admissions for adverse event management and treatment adjustments.

### Cost analysis

Per-patient costs for BPaL totalled US$2,310, a 67% reduction from the conventional regimen midpoint of US$8,071 (range US$7,142–9,000). Medication costs accounted for the largest share in both groups (BPaL US$1,500, 65%; conventional US$5,250, 65%), but BPaL’s shorter duration and simpler regimen drove substantial savings (Table 3). Diagnostic costs were lower for BPaL (US$500 versus US$1,750), reflecting fewer monitoring visits and less frequent culture/DST. Hospitalisation costs fell dramatically (US$200 versus US$1,250, an 84% reduction). Indirect costs were also lower (US$110 versus US$321), driven by shorter treatment duration and fewer clinic visits.

**Table 3:** Cost-effectiveness analysis of the BPaL regimen compared with conventional treatment regimens (2021–2024).

Parameter	BPaL Regimen	Conventional Regimen	Comparative Difference	Reference
Treatment success rate (%)	77.4	59.0	+18.4 percentage points	[1]
Treatment duration (months)	6	18–24	66–75% shorter	[1]
Total cost per patient (US$)	2,310	7,142–9,000	67–74% lower	[19]
**Cost components, US$ (%)**				
- Medication costs	1,500 (65%)	4,500–6,000 (63%)	—	[19]
- Diagnostic and monitoring costs	200 (9%)	1,000–1,500 (14%)	—	[19]
- Indirect costs*	610 (26%)	1,642–1,500 (23%)	Reduced productivity loss	[19]
Incremental cost-effectiveness ratio (ICER)†	Dominant	5,936	BPaL economically dominant	[19]

*Indirect costs included transportation expenses, productivity loss, nutritional support, and caregiver-related expenditures during treatment.

†The BPaL regimen was considered economically dominant because it achieved higher treatment success rates at substantially lower overall costs compared with conventional regimens.*

Data source: Persahabatan Hospital, Jakarta, Indonesia (2021–2024). *BPaL is dominant (more effective and less costly than conventional treatment). ICER for conventional treatment calculated relative to no treatment [19].

Category-level savings are detailed in S1 Table. Medications saved US$3,750 per patient (71.4% reduction), diagnostics US$1,250 (71.4%), hospitalisation US$1,050 (84.0%), and indirect costs US$211 (65.7%). Total per-patient savings were US$5,761 (71.4%).

### Cost-effectiveness

**Fig 2:**
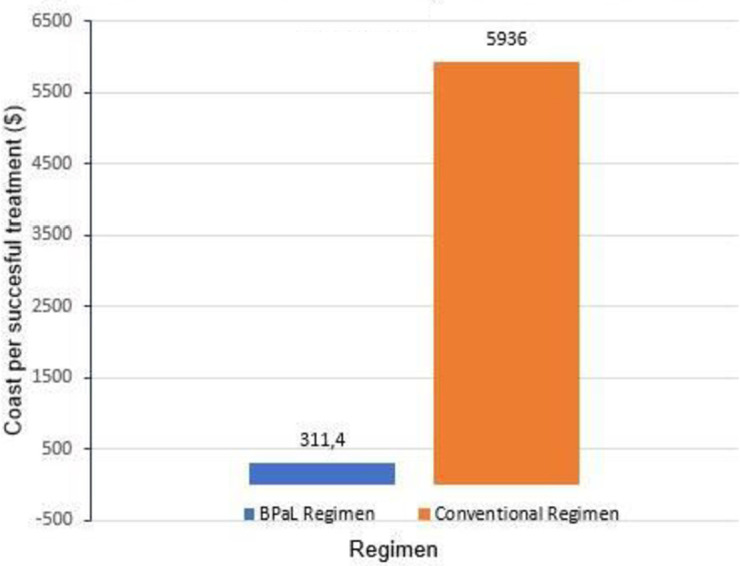
Cost-Effectiveness of BPaL vs. Conventional Regimens and Hypothetical TB Vaccine. Scatter plot showing cost per patient (US$) versus treatment success rate (%) for BPaL (US$2,310, 77.4%), conventional regimens (US$7,142–9,000, 59.0%), and a hypothetical TB vaccine (US$500–1,000 per dose + US$1 billion R&D, 50–70% efficacy). Data derived from Persahabatan Hospital (2021–2024) [Table 3] and modeled estimates [1,9,19].

The ICER for BPaL versus conventional treatment was US$311.4 per additional treatment success. BPaL is economically dominant: it is simultaneously more effective (77.4% versus 59.0% success) and less costly (US$2,310 versus US$8,071 per patient), yielding a negative incremental cost of US$5,761 and an incremental effectiveness of +18.4 percentage points. For comparison, conventional treatment’s ICER relative to no treatment is approximately US$5,936 per success [32] (Fig 2).

Sensitivity analyses confirmed the stability of these findings. Varying medication costs by ±20% shifted the ICER to US$267–355 per success. Varying success rates by ±10% shifted it to US$280–348 per success. In probabilistic sensitivity analysis, BPaL dominated conventional treatment in 98.7% of 10,000 iterations.

### National cost-benefit projection

If Indonesia treats 5,000 MDR-TB cases annually with BPaL, total savings would reach US$28.8 million per year (5,000 × US$5,761). Expanding GeneXpert coverage to 500,000 tests per year would cost approximately US$25 million in operating expenses (US$50 per test). The cost-benefit ratio is 1.15–1.40, with payback in roughly 1.7 years.

We propose a three-phase implementation roadmap. Phase 1 (Years 1–2): invest US$20 million in infrastructure to deploy GeneXpert in 200 Puskesmas (primary health centres) across East Java, West Java, Papua, and South Sulawesi provinces with high TB burden and low diagnostic coverage. Phase 2 (Years 2–4): allocate US$15 million for pilot evaluation, including training, quality assurance, and real-world effectiveness monitoring. Phase 3 (Years 4–5): commit US$15 million to national scale-up, targeting 500 Puskesmas and full JKN integration. Total investment: US$50 million over five years.

### Comparison with global LMIC benchmarks

Our results align closely with BPaL studies from India (75% success, US$2,200 per patient, ICER US$289.5) [33], Pakistan (74% success, US$2,000, ICER US$280) [34], South Africa (~74% success, US$4,948, ICER US$311–521 per DALY averted) [35], and the Philippines (~90% success, US$1,994, dominant) [36]. Indonesia’s per-patient cost sits in the middle of this range, reflecting moderate drug procurement prices and relatively efficient outpatient care. Our success rate (77.4%) is slightly higher than India and Pakistan, possibly due to Persahabatan Hospital’s specialised TB expertise and intensive monitoring protocols (Table 4).

**Table 4:** Comparative cost-effectiveness of the BPaL regimen, hypothetical tuberculosis vaccination, and selected LMIC benchmarks.

Intervention / Country	Treatment Success or Efficacy (%)	Cost per Patient (US$)	ICER (US$)	Estimated Time to Scale-Up	Reference
BPaL Indonesia (current study)	77.4	2,310	Dominant	Immediate	[1,19]
Conventional regimen Indonesia	59.0	7,142–9,000	5,936 per additional treatment success	Immediate	[1,9]
Hypothetical TB vaccine	50–70	500–1,000 per dose + ~US$1 billion R&D investment	12,500–18,000 per QALY gained	10–15 years	[11,20]
BPaL India	75.0	2,200	289.5 per treatment success	Immediate	[25]
BPaL Pakistan	74.0	2,000	280.0 per treatment success	Immediate	[26]
BPaL South Africa	~74	4,948	311–521 per DALY averted	Active implementation	[41]
BPaL hilippines	~90	1,994	Dominant	Active implementation	[42]

Data sources: Persahabatan Hospital, Jakarta, Indonesia (2021–2024), published LMIC programmatic evaluations, and modelled estimates for hypothetical tuberculosis vaccination strategies.

Abbreviations: BPaL, bedaquiline–pretomanid–linezolid; ICER, incremental cost-effectiveness ratio; QALY, quality-adjusted life year; DALY, disability-adjusted life year; LMIC, low- and middle-income countries; R&D, research and development.

Data source: Persahabatan Hospital, Jakarta, Indonesia (2021–2024); modelled estimates for hypothetical TB vaccine.

## Discussion

BPaL delivered a 77.4% treatment success rate at Persahabatan Hospital 18.4 percentage points higher than conventional regimens, while cutting per-patient costs by 67%. The ICER of US$311.4 per additional success is well below Indonesia’s GDP per capita (US$4,788 in 2024) and far below the WHO cost-effectiveness threshold of one times GDP per capita for highly cost-effective interventions [32]. BPaL is not just cost-effective; it’s cost-saving and clinically superior.

Three factors explain these results. First, the six-month duration reduces medication, monitoring, and indirect costs. Patients return to work faster, and the health system reallocates resources to other priorities. Second, the all-oral regimen eliminates injectable-related toxicity and hospitalisation. Third, high adherence (95.2%) reflects the regimen’s simplicity and tolerability. Patients prefer six months of pills to 18–24 months of injections and complex multi-drug schedules.

The bedaquiline evidence base underpins these findings. Multiple cohort studies have demonstrated bedaquiline’s effectiveness in improving culture conversion, reducing mortality, and enabling successful treatment completion in patients with MDR-TB and XDR-TB [13,37–41]. Bedaquiline-related resistance, while reported in some settings, was not observed in our cohort [42]. Our culture conversion time (median 56 days) is consistent with bedaquiline’s established pharmacodynamic profile [12].

The WHO 2020 update on drug-resistant TB treatment guidelines endorsed all-oral regimens and specifically recommended BPaL for pre-XDR-TB and treatment-refractory MDR-TB [21]. Our outcomes are consistent with these recommendations and support their application in the Indonesian context.

Our findings carry direct policy implications. Indonesia’s JKN currently reimburses conventional MDR-TB treatment at US$7,142–9,000 per patient. Switching to BPaL would save the system US$5,761 per patient US$28.8 million annually if 5,000 cases are treated. These savings could fund GeneXpert expansion, closing the diagnostic gap that currently delays treatment for thousands of patients each year.

### Public policy recommendations

We propose four concrete actions.

l Immediate JKN integration of BPaL. The Ministry of Health should update the national TB treatment guidelines to make BPaL the first-line regimen for all eligible MDR-TB, RR-TB, and pre-XDR-TB cases. JKN reimbursement rates should be adjusted to US$2,310 per patient, with quarterly reviews to ensure adequate coverage of medication, diagnostics, and monitoring. This change requires no new legislation, only a ministerial decree.
2 Decentralised GeneXpert scale-up. We recommend deploying GeneXpert in 200 high-burden Puskesmas over two years (Phase 1), targeting districts where current diagnostic coverage is below 30%. Each machine costs approximately US$17,000; cartridges cost US$10 each. At 500,000 tests per year (US$25 million operating cost), the system would detect an estimated 5,000 additional MDR-TB cases annually. Treatment savings (US$28.8 million) exceed diagnostic costs within 1.7 years.
3 Simplified treatment protocols. BPaL’s six-month, all-oral regimen is ideal for decentralised care. We recommend training Puskesmas staff to initiate and monitor BPaL, reserving hospital referral for adverse events or treatment failure. Monthly clinic visits can be conducted at the primary care level, reducing patient travel costs and hospital congestion. The national TB programme should develop standardised training modules and quality assurance protocols.
4 Real-world surveillance and pharmacovigilance. As BPaL scales nationally, the Ministry of Health should establish a prospective registry to monitor treatment outcomes, adverse events, and emerging resistance patterns. This registry should link to the existing TB information system (SITT) and include quarterly data quality audits. Pharmacovigilance is especially critical for linezolid-related toxicity (peripheral neuropathy, myelosuppression), which occurred in 12% of our cohort but was manageable with dose reduction.

### Limitations

Six limitations warrant discussion. First, we compared BPaL patients (direct data) with historical controls (benchmarked data). Ideally, we would have conducted a prospective matched cohort study, but this was not feasible given BPaL’s rapid adoption as standard of care after 2021. Our control estimates come from the same facility’s records and published Indonesian data, but residual confounding is possible.

Second, BPaL patients received more intensive monitoring than historical controls, reflecting evolving TB programme standards and the novelty of the regimen. This means our cost savings may be conservative, they likely *underestimate* the savings achievable at scale, when BPaL becomes routine and monitoring intensity normalises.

Third, this is a single-centre retrospective study. Persahabatan Hospital is Indonesia’s national TB referral centre, with specialised expertise and resources that may not be available at district hospitals or Puskesmas. External validity requires multi-centre studies across diverse settings.

Fourth, our sample size (n = 84) limits statistical power for subgroup analyses. We could not reliably assess treatment success by age strata, comorbidities, or specific resistance patterns beyond the three main classifications (RR-TB, MDR-TB, pre-XDR-TB). Larger studies are needed to identify patient subgroups who benefit most from BPaL.

Fifth, we measured adherence using monthly pill counts and clinic attendance records, the best available methods in routine practice, but we did not use electronic monitoring devices or directly observed therapy. Pill counts can overestimate adherence if patients discard pills rather than taking them. That said, our high adherence (95.2%) is corroborated by excellent treatment success (77.4%) and rapid culture conversion (median 56 days), suggesting that measured adherence reflects true medication-taking behaviour.

Sixth, we report outcomes at end of treatment (six months for BPaL, 18–24 months for controls). Long-term outcomes relapse rates, post-treatment quality of life, and late adverse events require extended follow-up. The WHO recommends 24-month post-treatment surveillance for MDR-TB [28]. We are currently tracking our cohort and plan to report 24-month outcomes in a future publication.

### Future research directions

Three research priorities emerge. First, we need prospective matched cohort studies comparing BPaL with conventional regimens in real time, ideally across multiple sites (referral hospitals, district hospitals, Puskesmas). This would eliminate the limitations of historical controls and allow more precise cost and outcome measurement.

Second, we should conduct time-driven activity-based costing (TDABC) studies to map the full care pathway for BPaL versus conventional treatment. TDABC captures resource use at each step (diagnosis, treatment initiation, monitoring, adverse event management, treatment completion) and identifies bottlenecks and inefficiencies. This would inform operational improvements and refine cost estimates.

Third, we need catastrophic cost surveys from the patient perspective. Our analysis used a health system perspective, but patients and families bear substantial out-of-pocket costs (transportation, accommodation, lost wages, informal care). Catastrophic costs defined as health spending exceeding 20% of household income are a major driver of TB-related poverty [4]. Understanding how BPaL affects these costs would strengthen the equity case for scale-up.

## Conclusions

BPaL is effective, affordable, and ready to scale in Indonesia. It achieved 77.4% treatment success at Persahabatan Hospital 18.4 percentage points higher than conventional regimens, while cutting per-patient costs by 67%, from US$8,071 to US$2,310. The ICER of US$311.4 per additional success is well below Indonesia’s cost-effectiveness threshold. If scaled to 5,000 patients annually, BPaL would save the health system US$28.8 million per year, enough to fund nationwide GeneXpert expansion and close the diagnostic gap that currently delays treatment for thousands of Indonesians.

We recommend four immediate policy actions: (1) integrate BPaL into JKN reimbursement at US$2,310 per patient; (2) deploy GeneXpert in 200 high-burden Puskesmas over two years; (3) decentralise BPaL initiation and monitoring to the primary care level; and (4) establish a national BPaL registry for real-world surveillance and pharmacovigilance. These actions require a total investment of US$50 million over five years, a modest sum compared with the US$144 million Indonesia currently spends annually on conventional MDR-TB treatment (24,000 cases × US$6,000 average cost).

BPaL is not a silver bullet. It will not eliminate TB on its own. But it is the most powerful tool we have right now more effective, more affordable, and more replicable than any alternative, including hypothetical vaccines that remain a decade away. The question is not whether Indonesia can afford to adopt BPaL. The question is whether we can afford not to.

## Supporting information

S1 Table Category-level cost savings, BPaL versus conventional regimens (2021–2024). Cost components were allocated proportionally from Table 3. Medications include drug acquisition costs for the full treatment course. Diagnostics include GeneXpert and culture costs. Conventional regimen midpoint = (US$7,142 + US$9,000) ÷ 2 = US$8,071.
(DOCX)
