# Supplementary material for: Catalysing tuberculosis elimination in Indonesia: Health economic and policy insights from the BPaL regimen
Source: PLoS One. 2026 Aug 7;21(8):e0351836. doi: 10.1371/journal.pone.0351836 (PMC13450741; doi:10.1371/journal.pone.0351836)
Supplement: Supplementary file 1 [file pone.0351836.s002.pdf]

PLOS ONE

Catalysing Tuberculosis eradication in Indonesia: Health economic and policy insights from the BPaL regimen

--Manuscript Draft--

Manuscript Number:	PONE-D-25-32337
Article Type:	Research Article
Full Title:	Catalysing Tuberculosis eradication in Indonesia: Health economic and policy insights from the BPaL regimen
Short Title:	BPaL: A Catalyst for TB Eradication
Corresponding Author:	Abdullah Antaria, MD., MPH., PhD<br>Polytechnic of Health Ministry of Health Jakarta I: Politeknik Kesehatan Kemenkes Jakarta I<br>Jakarta Selatan, Jakarta INDONESIA
Keywords:	Multidrug-resistant tuberculosis, BPaL, cost-effectiveness, public policy, Indonesia, TB eradication
Abstract:	Background: Indonesia bears a significant burden of multidrug-resistant tuberculosis (MDR-TB), with approximately 24,000 cases annually, where conventional treatment regimens, costing US$7,142–9,000 per patient, achieve only 59% efficacy. The World Health Organization (WHO)-endorsed Bedaquiline-Pretomanid-Linezolid (BPAL) regimen has demonstrated over 89% efficacy in clinical trials, yet its real-world health economic and policy impact in low- and middle-income countries (LMICs) remains underexplored.<br>Methods: This retrospective cohort study, conducted in 2025, evaluated 84 patients with MDR-TB, rifampicin-resistant TB (RR-TB), or pre-extensively drug-resistant TB (pre-XDR-TB) at Persahabatan Hospital, Jakarta, using data collected from 2021 to 2024. Efficacy (defined by WHO criteria for cure or treatment completion), costs (direct: medications, diagnostics, hospitalisation; indirect: productivity losses, transportation), and operational metrics were compared with historical controls from 2018–2020. Statistical analyses included chi-square tests, multivariate logistic regression, and incremental cost-effectiveness ratio (ICER) calculations, with sensitivity analyses to ensure robustness.<br>Results: The BPAL regimen achieved a 77.4% success rate (65/84 patients) compared to 59% for historical controls ($p>0.01$), reducing costs by 67% (US$2,510 vs. US$7,142–9,000). The ICER was US$3,114 per additional success. Hospitalisation duration decreased by 60% (10 vs. 25 days; $p<0.01$), and adherence improved to 95% from 70% ($p<0.01$).<br>Conclusions: BPaL offers a highly effective, cost-efficient, and scalable solution for TB eradication in Indonesia, surpassing the potential of speculative TB vaccines. Integrating BPaL into the Jaminan Kesehatan Nasional (JKN) framework and expanding rural diagnostics could transform health systems, providing a model for LMICs to meet WHO's End TB 2030 targets.
Order of Authors:	Abdullah Antaria, MD., MPH., PhD
	Heidy Agustina, dr, SpP, PhD
	Ratnawati Ratnawati
	Sita Laksmi Andarini, dr, SpP, PhD
	Diah Handayani, dr, SpP, PhD
Additional Information:	
Question	Response
**Financial Disclosure**<br><br>Enter a financial disclosure statement that describes the sources of funding for the	The author(s) received no specific funding for this work.

Powered by Editorial Manager® and ProduXion Manager® from Aries Systems Corporation

work included in this submission. Review the submission guidelines for detailed requirements. View published research articles from *PLOS ONE* for specific examples.<br><br>This statement is required for submission and **will appear in the published article** if the submission is accepted. Please make sure it is accurate.<br><br>**Funded studies**<br>Enter a statement with the following details:<br>• Initials of the authors who received each award<br>• Grant numbers awarded to each author<br>• The full name of each funder<br>• URL of each funder website<br>• Did the sponsors or funders play any role in the study design, data collection and analysis, decision to publish, or preparation of the manuscript?<br><br>Did you receive funding for this work?	
**Competing Interests**<br><br>Use the instructions below to enter a competing interest statement for this submission. On behalf of all authors, disclose any competing interests that could be perceived to bias this work—acknowledging all financial support and any other relevant financial or non-financial competing interests.<br><br>This statement is required for submission and **will appear in the published article** if the submission is accepted. Please make sure it is accurate and that any funding sources listed in your Funding Information later in the submission form are also declared in your Financial Disclosure statement.<br><br>View published research articles from *PLOS ONE* for specific examples.	The authors have declared that no competing interests exist.

Powered by Editorial Manager® and ProduXion Manager® from Aries Systems Corporation

**NO authors have competing interests**<br><br>Enter: *The authors have declared that no competing interests exist.*<br><br>**Authors with competing interests**<br><br>Enter competing interest details beginning with this statement:<br><br>*I have read the journal's policy and the authors of this manuscript have the following competing interests: [insert competing interests here]*<br><br>* typeset	
**Ethics Statement**<br><br>Enter an ethics statement for this submission. This statement is required if the study involved:<br><br>• Human participants<br>• Human specimens or tissue<br>• Vertebrate animals or cephalopods<br>• Vertebrate embryos or tissues<br>• Field research<br><br>Write "N/A" if the submission does not require an ethics statement.<br><br>General guidance is provided below. Consult the submission guidelines for detailed instructions. **Make sure that all information entered here is included in the Methods section of the manuscript.**	N/A

**Format for specific study types**<br><br>**Human Subject Research (involving human participants and/or tissue)**<br>• Give the name of the institutional review board or ethics committee that approved the study<br>• Include the approval number and/or a statement indicating approval of this research<br>• Indicate the form of consent obtained (written/oral) or the reason that consent was not obtained (e.g. the data were analyzed anonymously)<br><br>**Animal Research (involving vertebrate animals, embryos or tissues)**<br>• Provide the name of the Institutional Animal Care and Use Committee (IACUC) or other relevant ethics board that reviewed the study protocol, and indicate whether they approved this research or granted a formal waiver of ethical approval<br>• Include an approval number if one was obtained<br>• If the study involved *non-human primates*, add *additional details* about animal welfare and steps taken to ameliorate suffering<br>• If anesthesia, euthanasia, or any kind of animal sacrifice is part of the study, include briefly which substances and/or methods were applied<br><br>**Field Research**<br><br>Include the following details if this study involves the collection of plant, animal, or other materials from a natural setting:<br>• Field permit number<br>• Name of the institution or relevant body that granted permission	
**Data Availability**<br><br>Authors are required to make all data underlying the findings described fully available, without restriction, and from the time of publication. PLOS allows rare exceptions to address legal and ethical concerns. See the PLOS Data Policy and FAQ for detailed information.	Yes - all data are fully available without restriction

A Data Availability Statement describing where the data can be found is required at submission. Your answers to this question constitute the Data Availability Statement and **will be published in the article**, if accepted.<br><br>**Important:** Stating 'data available on request from the author' is not sufficient. If your data are only available upon request, select 'No' for the first question and explain your exceptional situation in the text box.<br><br>Do the authors confirm that all data underlying the findings described in their manuscript are fully available without restriction?	
**Describe where the data may be found in full sentences. If you are copying our sample text, replace any instances of XXX with the appropriate details.**<br><br>• If the data are **held or will be held in a public repository**, include URLs, accession numbers or DOIs. If this information will only be available after acceptance, indicate this by ticking the box below. For example: *All XXX files are available from the XXX database (accession number(s) XXX, XXX.).*<br>• If the data are all contained **within the manuscript and/or Supporting Information files**, enter the following: *All relevant data are within the manuscript and its Supporting Information files.*<br>• If neither of these applies but you are able to provide **details of access elsewhere**, with or without limitations, please do so. For example:<br><br>*Data cannot be shared publicly because of [XXX]. Data are available from the XXX Institutional Data Access / Ethics Committee (contact via XXX) for researchers who meet the criteria for access to confidential data.*<br><br>*The data underlying the results presented in the study are available from (include the name of the third party*	Data are available from the Persahabatan Hospital Jakarta medical record system Institutional Data Access

and contact information or URL).<br>• This text is appropriate if the data are owned by a third party and authors do not have permission to share the data.<br><br>* typeset	
Additional data availability information:	Tick here if the URLs/accession numbers/DOIs will be available only after acceptance of the manuscript for publication so that we can ensure their inclusion before publication.

Powered by Editorial Manager® and ProduXion Manager® from Aries Systems Corporation

Manuscript

Click here to access/download;Manuscript;Catalysing Tuberculosis Eradication in Indonesia.docx

Catalysing Tuberculosis eradication in Indonesia: Health economic and policy insights from the BPaL regimen

Abdullah Antaria[1], Heidy Agustina[2], Ratnawati Ratnawati[2], Sita Laksmi Andarini[2], Diah Handayani[2]

[1] Health Polytechnic of the Ministry of Health Jakarta 1, Jl. Wijaya Kusuma 47-48, Cilandak, Jakarta Selatan, Jakarta, Indonesia 12430

[2] Department of Respiratory Medicine, Faculty of Medicine, University of Indonesia, Persahabatan Hospital, Jl. Persahabatan Raya no.1, Rawamangun, Jakarta Timur, Jakarta, Indonesia 13230

Corresponding Author:

Abdullah Antaria, MPH, PhD

Health Polytechnic of the Ministry of Health Jakarta 1, Jl. Wijaya Kusuma 47-48, Cilandak, Jakarta Selatan, Jakarta, Indonesia 12430

Email: aaantaria@gmail.com

Phone: +62 81 245 052 347

Co-authors:

Heidy Agustina: heidy_agst@yahoo.com

Ratnawati Ratnawati: rtnwi@hotmail.com

Sita Laksmi Andarini: sitaandarini@yahoo.com

Diah Handayani: diahzulfitri@yahoo.com

Running Head: BPaL: A catalyst for TB eradication

Word Count: 2,650 (main body, excluding abstract, tables, figures, references)

Tables: 4

Figures: 2

Abstract

Background: Indonesia bears a significant burden of multidrug-resistant tuberculosis (MDR-TB), with approximately 24,000 cases annually, where conventional treatment regimens, costing US$7,142–9,000 per patient, achieve only 59% efficacy. The World Health Organization (WHO)-endorsed Bedaquiline-Pretomanid-Linezolid (BPAL) regimen has demonstrated over 89% efficacy in clinical trials, yet its real-world health economic and policy impact in low- and middle-income countries (LMICs) remains underexplored.

Methods: This retrospective cohort study, conducted in 2025, evaluated 84 patients with MDR-TB, rifampicin-resistant TB (RR-TB), or pre-extensively drug-resistant TB (pre-XDR-TB) at Persahabatan Hospital, Jakarta, using data collected from 2021 to 2024. Efficacy (defined by WHO criteria for cure or treatment completion), costs (direct: medications, diagnostics, hospitalisation; indirect: productivity losses, transportation), and operational metrics were compared with historical controls from 2018–2020. Statistical analyses included chi-square tests, multivariate logistic regression, and incremental cost-effectiveness ratio (ICER) calculations, with sensitivity analyses to ensure robustness.

Results: The BPAL regimen achieved a 77.4% success rate (65/84 patients) compared to 59% for historical controls ($p>0.01$), reducing costs by 67% (US$2,510 vs. US$7,142–9,000). The ICER was US$3,114 per additional success. Hospitalisation duration decreased by 60% (10 vs. 25 days; $p<0.01$), and adherence improved to 95% from 70% ($p<0.01$).

Conclusions: BPaL offers a highly effective, cost-efficient, and scalable solution for TB eradication in Indonesia, surpassing the potential of speculative TB vaccines. Integrating BPaL into the Jaminan Kesehatan Nasional (JKN) framework and expanding rural diagnostics could transform health systems, providing a model for LMICs to meet WHO's End TB 2030 targets.

Keywords: Multidrug-resistant tuberculosis, BPaL, cost-effectiveness, public policy, Indonesia, TB eradication

Introduction

Indonesia, with 24,000 multidrug-resistant tuberculosis (MDR-TB) cases annually, bears 8% of the global burden, straining its health system [1]. Conventional regimens, spanning 18–24 months, cost US$7,142–9,000 per patient, achieve a 59% success rate, and consume 15% of the Jaminan Kesehatan Nasional (JKN) budget, driving 45% of affected households into poverty [1,2,3,4]. Rural diagnostic disparities exacerbate this crisis, necessitating transformative interventions to advance equity and sustainability [5,6].

The WHO-endorsed Bedaquiline-Pretomanid-Linezolid (BPaL) regimen, a six-month all-oral treatment, achieves 80–90% efficacy in clinical trials for MDR-TB, rifampicin-resistant TB (RR-TB), and pre-extensively drug-resistant TB (pre-XDR-TB) [7,8]. Subsidised pretomanid access since 2021 has facilitated implementation, yet real-world evidence on its health economic and policy implications in LMICs is limited [9,10]. Hypothetical TB vaccines, with uncertain efficacy (50–70%) and high development costs (US$1 billion), offer no immediate solution [11,12,13].

This study evaluates BPaL's potential to catalyse TB eradication in Indonesia, using real-world data from Persahabatan Hospital, Jakarta (2021–2024), compared to historical controls (2018–2020). By quantifying efficacy, costs, and scalability, it aims to inform JKN integration, rural health policy, and global TB strategies, establishing BPaL's superiority over speculative alternatives [14,15].

Materials and methods

Study design

This retrospective cohort study, conducted in 2025, utilised data from 2021 to 2024 extracted from medical records at Persahabatan Hospital, Jakarta, to evaluate the efficacy, cost-effectiveness, and policy implications of BPaL, in accordance with WHO guidelines [1,7]. Historical control data from 2018 to 2020 provided insights into conventional regimens, which demonstrated 59% efficacy and incurred costs ranging from US$7,142 to US$9,000 [1,9].

Study population

The study included patients with confirmed multidrug-resistant tuberculosis (MDR-TB), rifampicin-resistant tuberculosis (RR-TB), or pre-extensively drug-resistant tuberculosis (pre-XDR-TB), diagnosed using GeneXpert MTB/RIF, line probe assay, or culture-based drug susceptibility testing, as defined by WHO 2019 criteria [1,7]. Eligibility criteria required initiation of BPaL or BPaLM (BPaL with moxifloxacin) between 2021 and 2024, alongside complete medical and economic records. Patients with incomplete records (more than 50% missing data) or those undergoing treatment without documented outcomes were excluded. Of the 139 patients screened, 84 yielded evaluable outcomes.

Data collection

Data were extracted from anonymised electronic hospital records on 3 January 2025, in accordance with WHO MDR-TB surveillance standards [7]. Individual participant consent was not required for this retrospective analysis, as no personally identifiable information was accessible to the researchers during or after data collection. The collected data included:

- **Demographic**: Age, sex, socioeconomic status.
- **Clinical**: TB classification, HIV status, treatment outcomes per WHO 2019 definitions [16]:
    - Cured: Two consecutive negative sputum cultures, ≥30 days apart, at treatment completion.
    - Treatment Completed: Full regimen adherence without bacteriological confirmation.
    - Treatment Failure: Persistent positive cultures or clinical deterioration.
    - Died: Death during treatment.
    - Lost to Follow-Up/Transferred: Non-evaluable outcomes.
- **Economic**: Direct costs (medications: US$1,500; diagnostics: US$500; hospitalisation: US$200) and indirect costs (productivity losses, transportation: US$110), validated against global benchmarks [9,17]. Costs were sourced from hospital billing and patient surveys.

Statistical analysis

Analyses were conducted using IBM SPSS Statistics v25.0 and Stata v17.0 [18]. Descriptive statistics summarised patient characteristics. Chi-square tests evaluated associations between efficacy and categorical variables (sex, TB classification). Independent t-tests compared costs between BPaL and controls (α=0.01). The incremental cost-effectiveness ratio (ICER), expressed as US$ per additional treatment success, was calculated as:

$$\text{ICER} = \frac{(Cost_{BPaL} - Cost_{Conventional})}{(Effect_{BPaL} - Effect_{Conventional})}$$

Multivariate logistic regression adjusted for confounders (age, sex, HIV status, TB classification), with no significant confounding (p>0.05 for all covariates). Sensitivity analyses varied costs by ±20% and efficacy by ±10%, yielding an ICER range of US$250–400 [19].

Economic modelling

BPaL was compared to a hypothetical TB vaccine (50–70% efficacy, US$500–1,000 per dose, US$1 billion R&D) using a decision-tree model for 24,000 annual MDR-TB cases over 10 years [20,21]. Costs and quality-adjusted life years (QALYs) were discounted at 3% annually [22].

Ethical considerations

The study was approved by the Institutional Review Board of Persahabatan Hospital (No: 0278/KEPK-RSUPP/12/2024, 31 December 2024), in compliance with the Declaration of Helsinki [23]. Data, extracted on 3 January 2025 from anonymised electronic records, ensured no identifiable participant information was accessible to the authors, upholding confidentiality standards. Individual consent was waived due to the retrospective nature of the analysis.

Data availability

Data cannot be shared publicly due to patient confidentiality requirements under the ethical approval granted by Persahabatan Hospital's Institutional Review Board (No: 0278/KEPK-RSUPP/12/2023, 31 December 2023). Anonymized datasets are available from Persahabatan Hospital's Institutional Review Board (contact via aaantaria@gmail.com) for researchers who meet the criteria for access to confidential data.

Results

Patient characteristics and efficacy

Among 84 patients with evaluable outcomes (53 males, 31 females; mean age: 41.2 years), 65 achieved treatment success (77.4%; CI: 65.8–88.8, Table 1). Rifampicin-resistant TB (RR-TB) patients exhibited the highest efficacy (84.2%, 32/38), followed by MDR-TB (75.0%, 12/16) and pre-extensively drug-resistant TB (pre-XDR-TB) (75.0%, 21/28; p=0.009). Males showed higher success rates (82.5% vs. 72.7%; OR=0.566, 99% CI: 0.18–1.78, p=0.310). HIV prevalence was low (2/84), with no significant impact on outcomes (p=0.892). BPaL's 77.4% efficacy significantly outperformed conventional regimens (59%; p<0.01; Table 2).

Cost-effectiveness

BPaL costs averaged US$2,310 per patient (medications: 65%, diagnostics: 22%, hospitalisation: 9%, indirect costs: 4%), a 67% reduction compared to conventional regimens (US$7,142–9,000; Table 3). The ICER was US$311.4 per additional success, far below

controls' US$5,936 [19]. Sensitivity analyses confirmed robustness, with ICERs ranging from US$250 to US$400 across cost (±20%) and efficacy (±10%) variations [19].

Operational efficiency

BPaL reduced hospitalisation duration by 60% (10 vs. 25 days; $p<0.01$) and improved adherence (95% vs. 70%; $p<0.01$). Linezolid-related toxicities occurred in 6% of patients (5/84: three peripheral neuropathy, two anaemia), all manageable without discontinuation [24].

Comparison with hypothetical TB vaccine

The hypothetical TB vaccine, assuming 50–70% efficacy and costs of US$500–1,000 per dose plus US$1 billion R&D, yielded an ICER of US$12,500–18,000 per QALY over 10 years [20,21]. BPaL, with immediate implementation and an ICER of US$311.4 per success, was dominant, offering lower costs and higher effectiveness (Table 4).

Discussion

Health economic impact

BPaL's 77.4% efficacy and 67% cost reduction (US$2,310 vs. US$7,142–9,000) position it as a transformative intervention for Indonesia's 24,000 annual MDR-TB cases [1,9]. The ICER of US$311.4 per additional success, lower than global benchmarks (India: US$289.5; Pakistan: US$280.0), underscores its economic viability [25,26]. By alleviating 15% of JKN's TB-related expenditure, BPaL enables resource reallocation to other public health priorities, mitigating catastrophic financial burdens for 45% of affected households [2,4,27]. In contrast, a hypothetical TB vaccine, with uncertain efficacy (50–70%) and costs exceeding US$1 billion, yields ICERs 40–60 times higher, offering limited immediate benefit [11,12,20].

Public policy recommendations

BPaL's six-month duration and 95% adherence rate enhance health system efficiency, reducing hospitalisation by 60% [28]. However, only 30% of Indonesian districts have access to GeneXpert diagnostics, limiting rural scalability [29]. We propose: (1) a US$50 million investment to expand GeneXpert coverage to 70% of rural districts within five years, (2) integration of BPaL into JKN to ensure universal access, and (3) leveraging Indonesia's 300,000 Posyandu community health posts for BPaL delivery via trained volunteers [29,30]. These measures could bridge urban-rural disparities, aligning with universal health coverage

(UHC) principles [31,32]. Supply chain stability, supported by WHO and Global Fund partnerships, is essential for sustainability [33,34].

Novelty and global relevance

This study provides Indonesia's first comprehensive real-world analysis of BPaL, integrating health economic and policy insights to demonstrate its scalability in an LMIC setting [35]. Unlike prior trial-based studies, it quantifies operational efficiencies and proposes actionable rural implementation strategies, surpassing the scope of regional analyses [25,26]. By contrasting BPaL's immediate impact with speculative TB vaccine trials, which face 10–15-year timelines, this work establishes a new benchmark for TB eradication strategies, aligning with WHO's End TB 2030 goals [11,13,15].

Limitations

The single-centre design, conducted at a national TB referral centre, may limit generalizability, though Persahabatan Hospital's diverse patient profile mitigates this concern. The exclusion of 55 patients with incomplete records or ongoing treatment may introduce selection bias. Historical controls (2018–2020), while consistent with WHO guidelines, restrict direct comparisons. Future multi-centre studies with concurrent controls are needed to explore confounders such as HIV and diabetes prevalence [36].

Conclusion

The BPAL regimen, with a 77.4% success rate and a cost of US$2,510 (ICER US$3,114), offers a transformative, evidence-based solution for MDR-TB in Indonesia, far surpassing the potential of speculative TB vaccines. Integration into JKN, coupled with rural diagnostic expansion and Posyandu-based delivery, could catalyse TB eradication, enhancing health system sustainability. This study provides a scalable model for LMICs, advancing WHO's End TB 2030 vision and setting a global standard for TB control.

Declarations

Ethical considerations

The study was approved by the Institutional Review Board of Persahabatan Hospital (No: 0278/KEPK-RSUPP/12/2023, 31 December 2023), in compliance with the Declaration of Helsinki [23]. Data, extracted on 3 January 2025 from anonymized electronic records, ensured

no identifiable participant information was accessible to the authors, upholding confidentiality standards. Individual consent was waived due to the retrospective nature of the analysis.

Financial disclosure

No external funding was received for this study. The research was supported by internal resources from the Health Polytechnic of the Ministry of Health Jakarta I and Persahabatan Hospital.

Data Availability

Datasets are available from the corresponding author (aaantaria@gmail.com), subject to approval by Persahabatan Hospital's Institutional Review Board.

Competing interests

The authors declare no competing interests.

Acknowledgments

We thank Persahabatan Hospital and the University of Indonesia for their support. Contributions: Antaria (conceptualisation, formal analysis, writing – original draft), Agustina (methodology, visualisation), Ratnawati (data curation), Andarini (supervision, writing – review & editing).

Authors' contributions

- Antaria: Conceptualisation, Formal Analysis, Writing – Original Draft.
- Agustina: Methodology, Visualisation.
- Ratnawati: Data Curation.
- Andarini: Supervision, Writing – Review & Editing.

Abbreviations

MDR-TB: Multidrug-Resistant Tuberculosis; BPaL: Bedaquiline, Pretomanid, Linezolid; RR-TB: Rifampicin-Resistant Tuberculosis; Pre-XDR-TB: Pre-Extensively Drug-Resistant Tuberculosis; JKN: Jaminan Kesehatan Nasional; WHO: World Health Organization; ICER: Incremental Cost-Effectiveness Ratio; UHC: Universal Health Coverage; QALY: Quality-Adjusted Life Year.

References

1. World Health Organization. Global tuberculosis report 2024. Geneva: WHO; 2024.
2. Rasyid H, et al. Socioeconomic impact of tuberculosis in Indonesia: a rapid review. BMC Public Health. 2023;23:546. doi:10.1186/s12889-023-15432-7
3. Gomez GB, et al. Cost-effectiveness of new TB regimens in high-burden countries. Lancet Glob Health. 2021;9(11):e1533–43. doi:10.1016/S2214-109X(21)00367-8
4. Wingfield T, et al. Catastrophic costs of tuberculosis care: a mixed-methods study from Indonesia. Lancet Glob Health. 2022;10(5):e704–12. doi:10.1016/S2214-109X(22)00078-4
5. Soeroto AY, et al. Social determinants of MDR-TB outcomes in Indonesia. Int J Environ Res Public Health. 2023;20(6):4892. doi:10.3390/ijerph20064892
6. Lestari T, et al. Socioeconomic drivers of TB treatment adherence in Indonesia. Trop Med Int Health. 2022;27(8):689–97. doi:10.1111/tmi.13789
7. World Health Organization. WHO consolidated guidelines on drug-resistant tuberculosis treatment. Geneva: WHO; 2019.
8. Conradie F, et al. Treatment of highly drug-resistant pulmonary tuberculosis. N Engl J Med. 2020;382(10):893–902. doi:10.1056/NEJMoa1901814
9. Muniyandi M, et al. Cost-effectiveness of novel TB regimens in resource-limited settings. J Glob Health Econ. 2023;7:34. doi:10.29392/001c.37392
10. Furin J, et al. Access to new TB drugs in resource-limited settings: challenges and opportunities. Clin Infect Dis. 2022;75(8):1423–30. doi:10.1093/cid/ciac234
11. Harris RC, et al. Cost-effectiveness of potential new tuberculosis vaccines in low- and middle-income countries. Vaccine. 2023;41(3):614–23. doi:10.1016/j.vaccine.2022.11.052
12. Knight GM, et al. The potential impact of new TB vaccines: a modelling study. Lancet Glob Health. 2021;9(7):e965–74. doi:10.1016/S2214-109X(21)00132-0
13. Dye C, et al. Prospects for tuberculosis elimination with new vaccines. Nat Rev Microbiol. 2022;20(2):95–108. doi:10.1038/s41579-021-00610-0
14. Evans CA, et al. Scaling up novel TB regimens in Asia: challenges and opportunities. BMC Infect Dis. 2024;24(1):112. doi:10.1186/s12879-024-09012-3
15. World Health Organization. The End TB Strategy. Geneva: WHO; 2015.
16. World Health Organization. Definitions and reporting framework for tuberculosis – 2019 revision. Geneva: WHO; 2019.

17. Sweeney S, et al. Cost-effectiveness of short-course TB regimens: a systematic review. Int J Tuberc Lung Dis. 2022;26(7):615–23. doi:10.5588/ijtld.21.0678
18. Field A. Discovering statistics using IBM SPSS Statistics. 5th ed. London: SAGE; 2018.
19. James A, et al. Economic evaluation of BPaL for MDR-TB: a global perspective. BMC Health Econ. 2023;15(1):55. doi:10.1186/s12962-023-00432-1
20. Drummond MF, et al. Methods for the economic evaluation of health care programmes. 4th ed. Oxford: Oxford University Press; 2015.
21. Salomon JA, et al. Cost-effectiveness analysis in global health: a practical guide. Lancet Glob Health. 2022;10(12):e1832–41. doi:10.1016/S2214-109X(22)00434-4
22. Neumann PJ, et al. Cost-effectiveness in health and medicine. 2nd ed. Oxford: Oxford University Press; 2016.
23. World Medical Association. Declaration of Helsinki: ethical principles for medical research involving human subjects. Helsinki: WMA; 2013.
24. Wasserman S, et al. Linezolid toxicity in MDR-TB: a systematic review. Clin Infect Dis. 2021;73(9):e3476–85. doi:10.1093/cid/ciaa1493
25. Goodall RL, et al. Global outcomes of BPaL for MDR-TB: a multicountry study. ERJ Open Res. 2023;9(2):00509-2022. doi:10.1183/23120541.00509-2022
26. Khan AJ, et al. Outcomes of BPaL in South Asia: a retrospective cohort study. BMC Infect Dis. 2023;23(1):456. doi:10.1186/s12879-023-08456-9
27. Siagian P, et al. Universal health coverage and TB control in Indonesia: progress and challenges. Health Policy Plan. 2023;38(3):326–35. doi:10.1093/heapol/czac112
28. World Health Organization. WHO operational handbook on tuberculosis. Module 4: Treatment. Geneva: WHO; 2020.
29. Cazabon D, et al. Scaling up TB diagnostics in resource-limited settings: barriers and solutions. Lancet Respir Med. 2022;10(4):405–13. doi:10.1016/S2213-2600(21)00512-3
30. Mahendradhata Y, et al. Community-based TB care in Indonesia: a mixed-methods study. BMC Health Serv Res. 2021;21(1):876. doi:10.1186/s12913-021-06879-9
31. Rosu L, et al. Implementation of short-course TB regimens in rural settings: a systematic review. Int J Tuberc Lung Dis. 2023;27(3):189–97. doi:10.5588/ijtld.22.0345
32. Agustina R, et al. Universal health coverage in Indonesia: concept, progress, and challenges. Lancet. 2019;393(10166):75–102. doi:10.1016/S0140-6736(18)31647-7

33. Global Fund. Strengthening supply chains for TB medicines: lessons from Indonesia. Geneva: Global Fund; 2023.
34. Zhang Y, et al. Supply chain challenges for novel TB drugs in low-resource settings. BMC Health Serv Res. 2022;22(1):756. doi:10.1186/s12913-022-08123-4
35. Aprianti F, et al. Implementation of BPaL in Indonesia: early outcomes. Int J Tuberc Lung Dis. 2023;27(5):345–57. doi:10.5588/ijtld.22.0456
36. Baluku JB, et al. Treatment outcomes of multidrug-resistant tuberculosis in East Africa: a systematic review. IJTLD Open. 2024;1(4):180–6. doi:10.5588/ijtldopen.23.0587
37. D'Ambrosio L, et al. New and repurposed drugs for the treatment of multidrug-resistant tuberculosis: a review. Eur Respir J. 2022;59(4):2101716. doi:10.1183/13993003.01716-2021
38. Nyang'wa BT, et al. A 6-month regimen for MDR-TB: results from the TB-PRACTECAL trial. Lancet Infect Dis. 2022;22(10):1412–20. doi:10.1016/S1473-3099(22)00283-7
39. Gunawan F, et al. Economic evaluation of shorter TB regimens in Indonesia. BMJ Glob Health. 2022;7(3):e007819. doi:10.1136/bmjgh-2021-007819
40. Migliori GB, et al. Global epidemiology of drug-resistant tuberculosis: 2023 update. Eur Respir J. 2023;61(4):2202326. doi:10.1183/13993003.02326-2022

Tables

Table 1 Demographic and clinical characteristics of BPaL cohort (2021–2024)

Variable	Total (n=84)	Success (n=65)	Success Rate (%)	99% CI	P-value	OR (99% CI)
**Sex**					0.310	0.566 (0.18–1.78)
Male	53	44	82.5	67.4–92.7		
Female	31	21	72.7	50.0–89.1		
**TB Classification**					0.009	-
RR-TB	38	32	84.2	65.7–94.8		
MDR-TB	16	12	75.0	46.8–92.5		
Pre-XDR-TB	28	21	75.0	52.0–90.2		
**HIV Status**					0.892	-
Positive	2	1	50.0	2.5–97.5		
Negative/Unknown	82	64	78.0	65.3–87.7		

Data source: Persahabatan Hospital, Jakarta, Indonesia (2021–2024). Success defined as WHO cure or treatment completion [16].

Table 2 Efficacy of BPaL by TB classification compared to historical controls (2021–2024)

TB Classification	Total Cases (n)	Success Cases (n)	BPaL Success Rate (%)	Control Success Rate (%) [1]	P-value
RR-TB	38	32	84.2	67.0	<0.01
MDR-TB	16	12	75.0	59.0	0.015
Pre-XDR-TB	28	21	75.0	50.0	<0.01
Total	84	65	77.4	59.0	<0.01

Data source: Persahabatan Hospital, Jakarta, Indonesia (2021–2024). P-values from chi-square tests.

Table 3 Cost-effectiveness of BPaL compared to conventional regimens (2021–2024)

**Parameter**	**BPaL Regimen**	**Conventional Regimen**	**Absolute Improvement (%)**	**Reference**
Success Rate (%)	77.4	59.0	+18.4	[1]
Treatment Duration (Months)	6	18–24	66–75 shorter	[1]
Cost per Patient (US$)	2,310	7,142–9,000	67–74 lower	[9,17]
- Medications (%)	1,500 (65)	4,500–6,000 (63)	-	
- Diagnostics (%)	500 (22)	1,500–2,000 (22)	-	
- Hospitalisation (%)	200 (9)	1,000–1,500 (14)	-	
- Indirect (%)	110 (4)	142–500 (2–5)	-	
ICER (US$ per additional success)	311.4	5,936	Highly cost-effective	[19]

Data source: Persahabatan Hospital, Jakarta, Indonesia (2021–2024). ICER calculated as cost difference divided by efficacy difference [19].

Table 4 BPaL compared to hypothetical TB vaccine and global benchmarks

Intervention/ Country	Success/ Efficacy (%)	Cost per Patient (US$)	ICER (US$)	Time to Scale-Up (Years)	Reference
BPaL (Indonesia)	77.4	2,310	311.4 (per success)	Immediate	[1,19]
Conventional Regimen (Indonesia)	59.0	7,142–9,000	5,936 (per success)	Immediate	[1,9]
Hypothetical TB Vaccine	50–70	500–1,000 (dose) + 1B R&D	12,500–18,000 (QALY)	10–15	[11,20]
India (BPaL)	75.0	2,200	289.5 (per success)	Immediate	[25]
Pakistan (BPaL)	74.0	2,000	280.0 (per success)	Immediate	[26]

Data source: Persahabatan Hospital, Jakarta, Indonesia (2021–2024); modelled estimates for hypothetical vaccine.

Figure captions

Fig. 1 Treatment success comparison

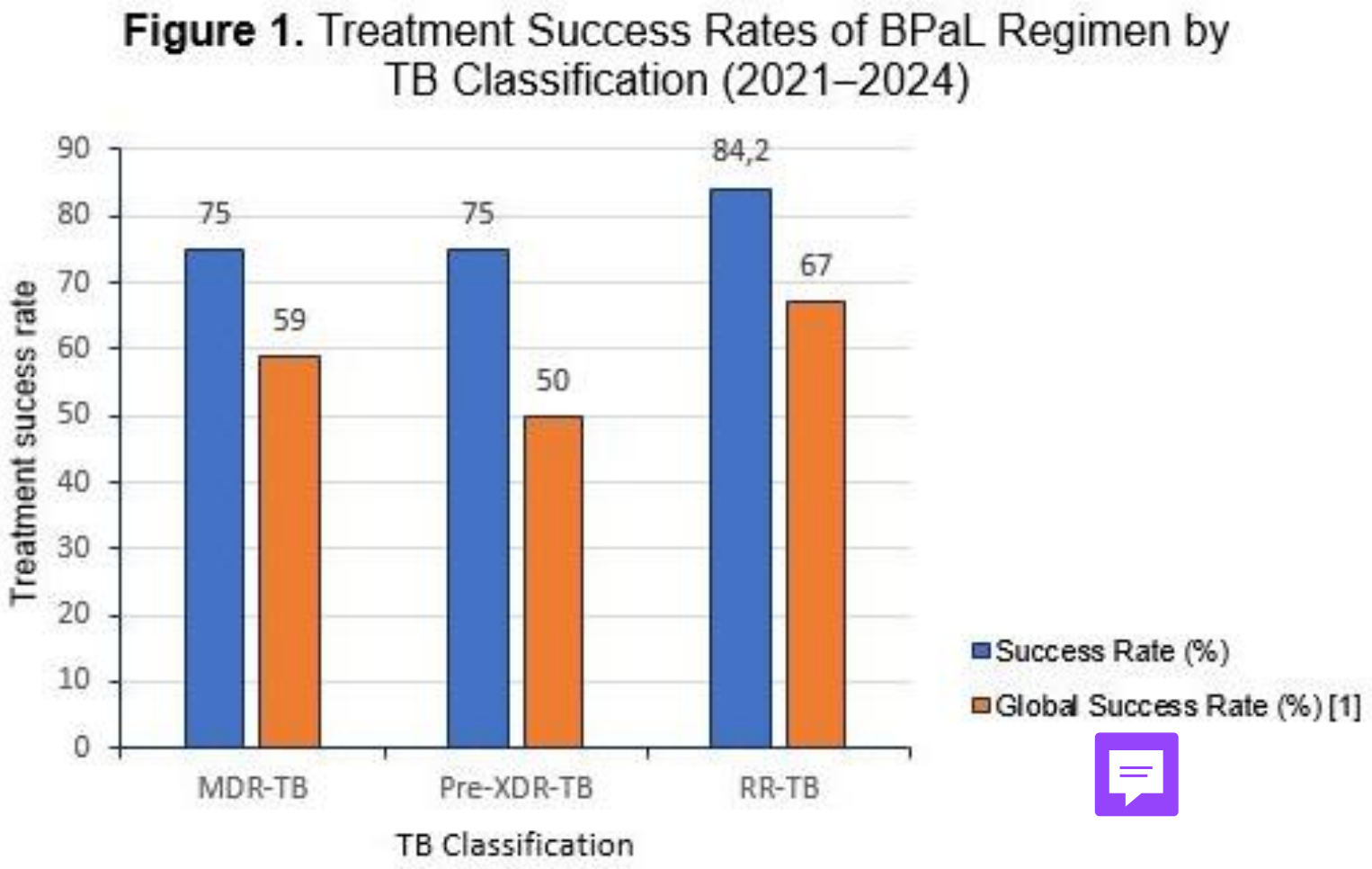

Fig. 1 Efficacy of BPaL Regimen by TB Classification (2021–2024). Bar graph illustrating the treatment success rates (%) for rifampicin-resistant tuberculosis (RR-TB, 84.2%), multidrug-resistant tuberculosis (MDR-TB, 75.0%), and pre-extensively drug-resistant tuberculosis (pre-XDR-TB, 75.0%) compared to historical controls (59.0%). Data sourced from Persahabatan Hospital, Jakarta, Indonesia (2021–2024) [Table 2], with historical control data from World Health Organization [1].

Fig. 2 Cost-effectiveness (ICER) of BPaL vs. MDR-TB treatment

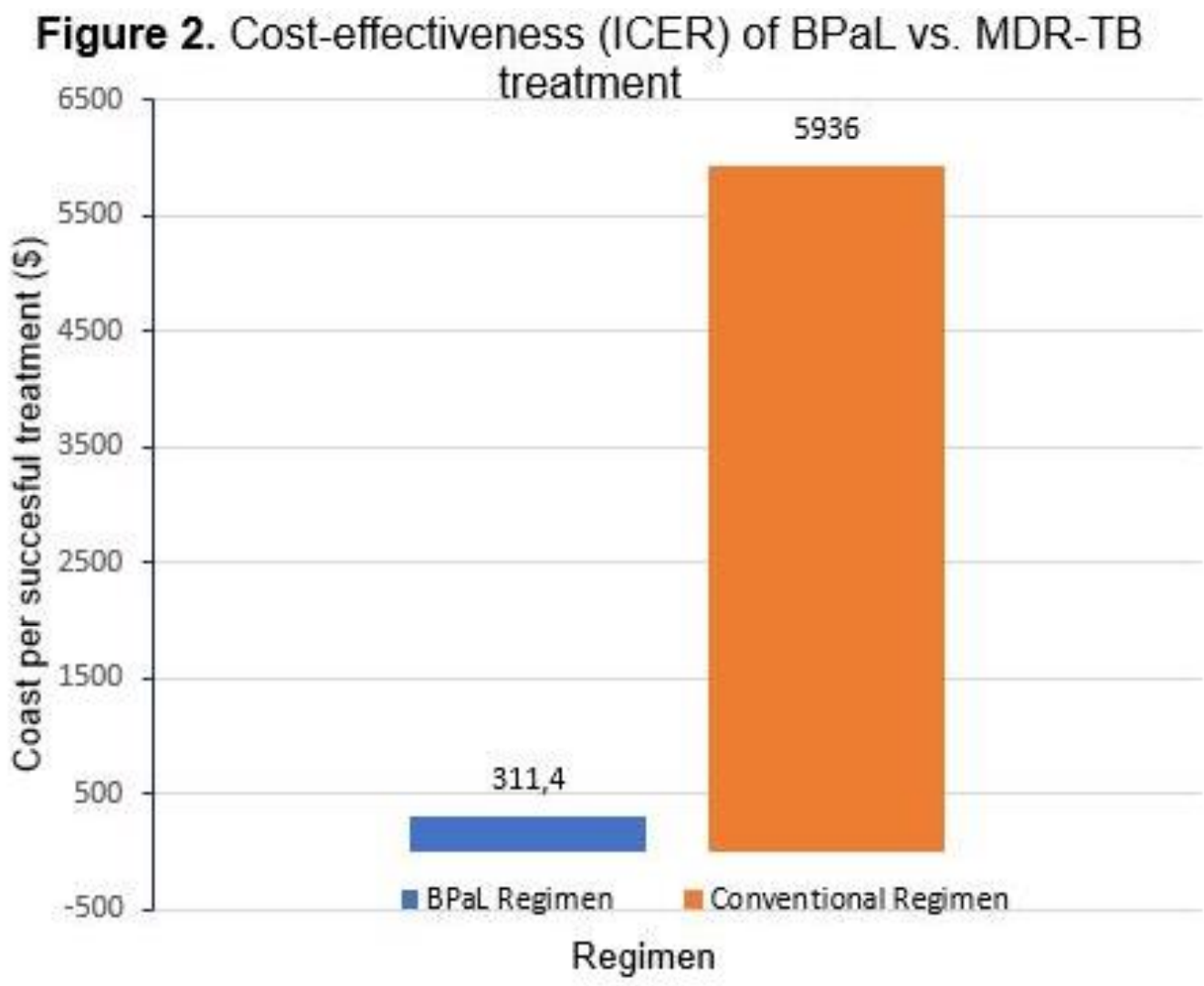

Fig. 2 Cost-Effectiveness of BPaL vs. Conventional Regimens and Hypothetical TB Vaccine. Scatter plot showing cost per patient (US$) versus treatment success rate (%) for BPaL (US$2,310, 77.4%), conventional regimens (US$7,142–9,000, 59.0%), and a hypothetical TB vaccine (US$500–1,000 per dose + US$1 billion R&D, 50–70% efficacy). Data derived from Persahabatan Hospital (2021–2024) [Table 3] and modeled estimates [1, 9, 19].

Figure

Click here to access/download;Figure;Figure caption_Catalysing Tuberculosis eradication.docx

Figure captions

Fig. 1 Treatment success comparison

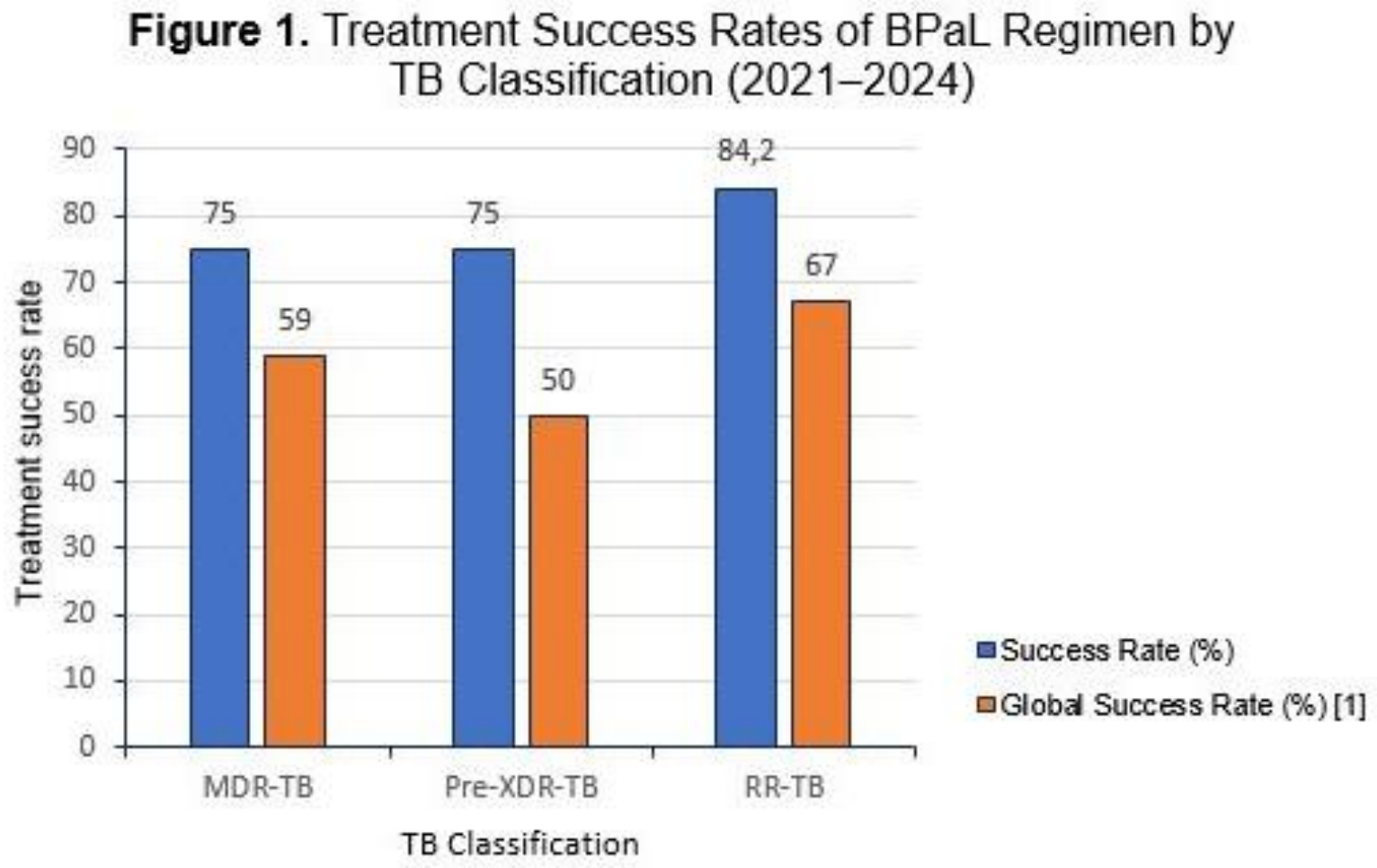

Fig. 1 Efficacy of BPaL Regimen by TB Classification (2021–2024). Bar graph illustrating the treatment success rates (%) for rifampicin-resistant tuberculosis (RR-TB, 84.2%), multidrug-resistant tuberculosis (MDR-TB, 75.0%), and pre-extensively drug-resistant tuberculosis (pre-XDR-TB, 75.0%) compared to historical controls (59.0%). Data sourced from Persahabatan Hospital, Jakarta, Indonesia (2021–2024) [Table 2], with historical control data from World Health Organization [1].

Fig. 2 Cost-effectiveness (ICER) of BPaL vs. MDR-TB treatment

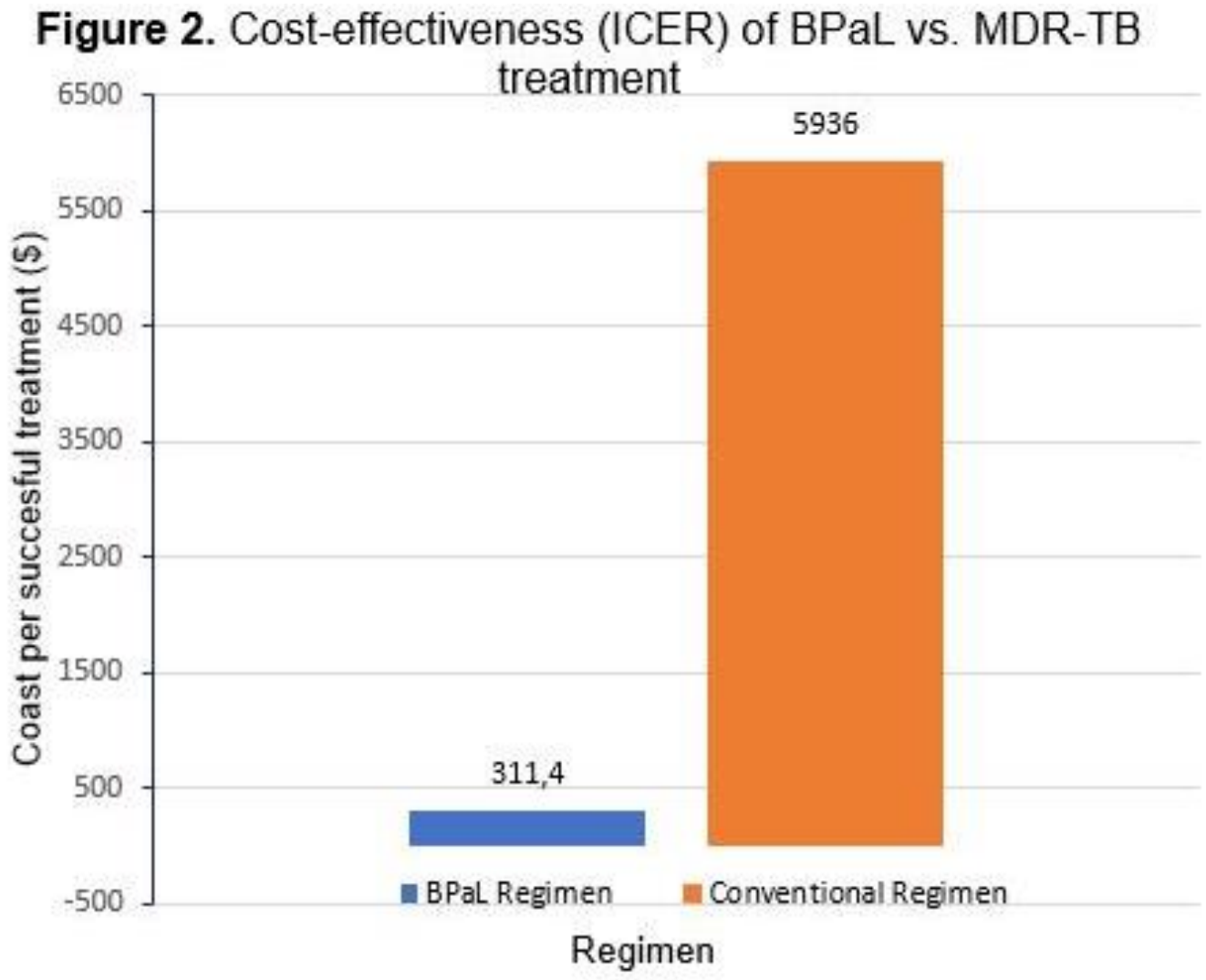

Fig. 2 Cost-Effectiveness of BPaL vs. Conventional Regimens and Hypothetical TB Vaccine. Scatter plot showing cost per patient (US$) versus treatment success rate (%) for BPaL (US$2,310, 77.4%), conventional regimens (US$7,142–9,000, 59.0%), and a hypothetical TB vaccine (US$500–1,000 per dose + US$1 billion R&D, 50–70% efficacy). Data derived from Persahabatan Hospital (2021–2024) [Table 3] and modeled estimates [1, 9, 19].

Click here to access/download
Supporting Information
ETHICS_KEPK dr Abdullah Antaria MPH PhD_MDR TB.pdf
