# Supplementary material for: Catalysing tuberculosis elimination in Indonesia: Health economic and policy insights from the BPaL regimen
Source: PLoS One. 2026 Aug 7;21(8):e0351836. doi: 10.1371/journal.pone.0351836 (PMC13450741; doi:10.1371/journal.pone.0351836)
Supplement: Supplementary file 2 [file pone.0351836.s001.docx]

**S1 Table.**

Category-level cost savings, BPaL versus conventional regimens (2021–2024).

| **Cost Category** | **BPaL (US$)** | **Conventional (US$)** | **Absolute Saving (US$)** | **Relative Saving (%)** |
| --- | --- | --- | --- | --- |
| Medications | 1,500 | 5,250 | 3,750 | 71.4 |
| Diagnostics | 500 | 1,750 | 1,250 | 71.4 |
| Hospitalisation | 200 | 1,250 | 1,050 | 84.0 |
| Indirect costs* | 110 | 321 | 211 | 65.7 |
| **Total** | **2,310** | **8,071** | **5,761** | **71.4** |
|  |  |  |  |  |

*Indirect costs include productivity losses, transportation expenses, nutritional support, and caregiver-related expenditures during the treatment period.

Conventional regimen midpoint = (US$7,142 + US$9,000) ÷ 2 = US$8,071. Cost components were allocated proportionally from Table 3 of the main manuscript. Medications include drug acquisition costs for the full treatment course. Diagnostics include GeneXpert, culture/DST, routine monitoring laboratories, and radiology. Hospitalisation includes bed-days, procedures, and adverse event-related inpatient care. All costs are reported in 2024 US dollars.
