# Supplementary material for: Catalysing tuberculosis elimination in Indonesia: Health economic and policy insights from the BPaL regimen
Source: PLoS One. 2026 Aug 7;21(8):e0351836. doi: 10.1371/journal.pone.0351836 (PMC13450741; doi:10.1371/journal.pone.0351836)
Supplement: Supplementary file 3 [file pone.0351836.s004.docx]

1. **Response to reviewers**

**Date:** 18 August 2025

**To	:** Gilbert Sterling Octavius, Academic Editor, PLOS ONE

**Subject	:** Revision of manuscript PONE-D-25-32337: “Catalysing tuberculosis elimination in Indonesia: Health economic and policy insights from the BPaL regimen”

**Dear Dr. Octavius and reviewers,**

We sincerely appreciate the thorough and insightful feedback on our manuscript. Your comments have significantly enhanced its scientific rigour and clarity. Below, we address each point raised by the academic editor and reviewers (Reviewer #1: Dr. Salah Foraida; Reviewer #2: Anonymous), outlining how revisions align with PLOS ONE’s publication criteria. The submission includes a marked-up manuscript (“Revised Manuscript with Track Changes”), an unmarked version (“Manuscript”), an updated cover letter with financial disclosure, and figure files verified via the PACE tool. We also address journal requirements (style, data availability, ethics, protocols.io) to ensure compliance.

**Response to journal requirements**

1. **PLOS ONE style requirements**

The manuscript adheres to PLOS ONE’s style templates (main body, title/authors/affiliations), with double-spaced text, line numbering, and British English. Files are named as required: “Revised Manuscript with Track Changes.docx,” “Manuscript.docx,” “Response to Reviewers.docx,” “Cover Letter.docx,” “Figure_1.jpeg,” and “Figure_2.jpeg.”

2. **Data availability statement**

Owing to ethical restrictions concerning patient confidentiality, as mandated by the Institutional Review Board (IRB) approval of Persahabatan Hospital (Reference No: 0278/KEPK-RSUPP/12/2024), the dataset cannot be made publicly available. To ensure sustained data access for interested researchers, requests for anonymised data may be directed to the Sub-Monitoring and Evaluation, Clinical Research Unit (ICRU) at Persahabatan Hospital. Contact details for the ICRU are as follows:

• Contact Person: Sumedi, PhD

• Email: medicrurspersahabatan@gmail.com

• Telephone: +62 852 1795 8182

Upon receipt of a formal request, the ICRU will provide the minimal anonymised dataset required to facilitate research reproducibility, in full compliance with applicable ethical and legal standards, including Indonesia’s data protection regulations. This approach aligns with PLOS ONE’s Data Availability Policy (http://journals.plos.org/plosone/s/data-availability). We welcome editorial guidance should further clarification or an exemption be necessary.

3. **Ethics statement**

The ethics statement has been relocated to the “Materials and Methods” section under “Ethical Considerations” and removed from other sections, detailing IRB approval and consent waiver per the Declaration of Helsinki [23].

4. **Suggested references**

We have incorporated the suggested references ([41]: https://doi.org/10.1371/journal.pone.0309034; [42]: https://doi.org/10.5588/ijtldopen.24.0094) in the discussion, as detailed below, enhancing the study’s global context.

**Response to reviewer #1 (Dr. Salah Foraida)**

1. **Terminology: “Eradication” vs. “Elimination”**

**Comment**: Replace “eradication” with “elimination” as it pertains to one country.

**Response**: We have replaced “eradication” with “elimination” throughout the manuscript (title, abstract, introduction, discussion, conclusion) to align with WHO’s definition of reducing TB incidence to zero (or <1 case per million) in a single country [15]. The revised title is “Catalysing Tuberculosis Elimination in Indonesia…”. Changes are tracked in the revised manuscript.

2. **Clarity of comparison to hypothetical TB vaccine**

**Comment**: The comparison to a hypothetical TB vaccine lacks clarity in the abstract, introduction, or methods sections.

**Response**: We added one to two sentences in the abstract and introduction (e.g., “To contextualise BPaL’s immediate impact, we modelled its cost-effectiveness against a hypothetical TB vaccine using conservative assumptions”). A new subsection, “Vaccine Comparison Model,” in the “Materials and Methods” details assumptions (50–70% efficacy [11]), software (Stata v17.0), and probabilistic sensitivity analysis for 24,000 annual MDR-TB cases over 10 years [20,21]. Changes are tracked.

3. **Use of WHO definitions for DR-TB and treatment outcomes**

**Comment**: Why use 2019 WHO definitions instead of the 2021 definitions?

**Response**: We updated to the 2021 WHO definitions [16a], which relax “cured” criteria to two negative cultures ≥7 days apart and include pre-treatment losses. Re-analysis confirmed no change to the 77.4% success rate. This is noted in the “Data Collection” subsection and “Limitations,” with [16a] added to the reference list. Changes are tracked.

4. **Figure 1: Clarification of labels and key**

**Comment**: Replace “global” with “historical or conventional” in Figure 1 and add “BPaL success rate” to the key.

**Response**: We replaced “global” with “historical controls” in Figure 1’s caption and key, clarifying Indonesia-specific data (2018–2020) [1]. “BPaL Success Rate” is added to the key with high-contrast colours (blue for BPaL, grey for controls). The figure is a 300 dpi JPEG, verified via PACE. Changes are tracked.

**Response to reviewer #2 (Anonymous)**

**General comment**: Congratulations to Persahabatan Hospital and the University of Indonesia for conducting this research, key to updating guidelines and ensuring cost-effective treatment.

**Response**: We thank Reviewer #2 for recognising the study’s policy relevance. Your feedback has guided our revisions to enhance clarity and impact.

1. **Different methods for data collection**

**Comment**: Data collection methods for BPaL and other regimens should be consistent, i.e., from hospital records in the same timeframe.

**Response**: We clarified in the “Study Design” section that historical controls (2018–2020) primarily use Persahabatan Hospital records, supplemented by WHO benchmarks [1,9] for <10% of cases. A sensitivity analysis in “Statistical Analysis” varies data sources, confirming robust ICERs (US$250–400). This limitation is noted in “Limitations,” recommending prospective studies [41]. Changes are tracked.

2. **Specification of Compared Regimens and Cost Calculation**

**Comment**: Specify regimens (short or long) and cost calculation methods.

**Response**: The “Study Design” section now specifies controls as long regimens (18–24 months, SLOR-like), with costs (US$7,142–9,000) as a weighted average from hospital billing and WHO benchmarks [1,9,41,42]. Short regimens (9–11-month SSOR, US$4,500–6,000) are noted, with BPaL’s savings potentially lower (36–50%) [42]. Changes are tracked.

3. **Reference to Suggested Papers**

**Comment**: Reference https://doi.org/10.1371/journal.pone.0309034 and <https://doi.org/10.5588/ijtldopen.24.0094>.

**Response**: Added [41] and [42] in the “Discussion” to benchmark BPaL’s ICER (US$311.4) against South Africa (US$311–521) and the Philippines (US$1,994 per success). This highlights Indonesia’s rural diagnostic challenges [29]. Changes are tracked.

4. **Impact of long time period on costs**

**Comment**: Specify the cost basis, given changing drug costs.

**Response**: The “Data Collection” section clarifies costs in 2024 USD, adjusted for inflation and drug price reductions (54% for bedaquiline, 25% for pretomanid post-2023 [15,19]). A time-trend sensitivity analysis in “Statistical Analysis” confirms sustained savings (ICER: US$250–400) [39]. Changes are tracked.

5. **Operational research vs. Programmatic use**

**Comment**: Clarify if analysis reflects operational research (OR) or programmatic use, as monitoring costs differ.

**Response**: The “Study Population” section specifies OR conditions (LIFT-TB, 2021–2024) with higher monitoring costs. “Data Collection” estimates programmatic costs as 15–25% lower [41,42]. This is noted in “Limitations” and sensitivity analyses. Changes are tracked.

6. **Catastrophic costs analysis**

**Comment**: Include analysis of patient-level catastrophic costs, if feasible.

**Response**: Limited household income data prevented a full analysis (>20% income). The “Discussion” notes BPaL’s low indirect costs (US$110) likely reduce catastrophic costs by 30–50%, as in South Africa (0% for BPaL vs. 20% for SLOR) [41]. This is flagged in “Limitations,” recommending WHO’s TB cost survey tool [42]. Changes are tracked.

**Additional revisions**

- **Competing interests**: Added: “The authors declare no competing interests.”
- **Tables and Figures**: Ensured self-explanatory with defined units and abbreviations.
- **Supporting information**: Included “S1_Dataset.xlsx” with aggregated statistics (e.g., patient demographics, costs) to support reproducibility while respecting IRB constraints.
- **Protocols.io**: Evaluated deposition; standard WHO protocols [1,7] are detailed in “Materials and methods.” We offer to deposit data extraction protocols if required.
- **Figure compliance**: Figures 1 and 2 are 300 dpi JPEGs, verified via PACE, with accessible captions.

We believe these revisions address all concerns, ensuring technical soundness, transparency, and policy relevance. We thank Dr. Foraida and Reviewer #2 for their valuable feedback and are prepared to address further queries to meet the 29 September 2025 deadline.

Sincerely,
dr. Abdullah Antaria, MPH, PhD

On behalf of all authors

Health Polytechnic of the Ministry of Health Jakarta 1

Jl. Wijaya Kusuma 47-48, Cilandak, Jakarta Selatan, Jakarta, Indonesia 12430

Email: [aaantaria@gmail.com](mailto:aaantaria@gmail.com)

Phone: +62 81 245 052 347
