# Supplementary material for: Catalysing tuberculosis elimination in Indonesia: Health economic and policy insights from the BPaL regimen
Source: PLoS One. 2026 Aug 7;21(8):e0351836. doi: 10.1371/journal.pone.0351836 (PMC13450741; doi:10.1371/journal.pone.0351836)
Supplement: Supplementary file 4 [file pone.0351836.s005.docx]

# Response to Reviewers

Manuscript ID: PONE-D-25-32337R1

Title: Catalysing tuberculosis elimination in Indonesia: Health economic and policy insights from the BPaL regimen

**Journal: PLOS ONE**

Date: 11 May 2026

Dr. Ilse Bloom

Staff Editor

PLOS ONE

Public Library of Science

Dear Dr. Bloom,

Thank you for your letter of 2 April and for the opportunity to revise the manuscript. Dr. Foraida and Reviewer #2 have confirmed that their earlier comments were satisfactorily addressed. We have now attended to the new point raised by Reviewer #2 and the four substantive comments from Reviewer #3.

Below we respond to each comment in turn. The marked-up version shows all changes. The core findings remain unchanged: 77.4% treatment success in 84 evaluable patients at Persahabatan Hospital, at US$2,310 per patient, a 67% cost reduction versus conventional regimens, with an ICER of US$311.4 per additional treatment success.

## Response to Reviewer #1 (Dr. Salah Foraida)

Reviewer comment:

“Thanks for addressing all of my previous comments. I hope this will provide a model to benefit DR-TB patients in Low and Middle Income Countries similar to Indonesia.”

Response:
Thank you, Dr. Foraida. That is exactly what we set out to do—provide an evidence-based, replicable model for other LMICs. Your engagement throughout the review process has been helpful.

*No further revisions requested. No changes made.*

## Response to Reviewer #2 (Anonymous)

Reviewer comment:

“Thank you for taking the time to respond to the queries and comments. The Limitations section, line 209: I believe there may be a typo error where it says ‘may overestimate savings;’ should say underestimate saving with BPaL. Apart from this, the article looks good.”

Response:
You are absolutely right. This was a directional error, and we are glad you caught it.

Change made: In the Limitations section, “may overestimate savings” has been replaced with “may underestimate the savings achievable at scale.”

Rationale: The BPaL cohort was conducted under operational research (LIFT-TB) conditions, which involve higher monitoring intensity and greater resource input than routine programmatic care. This means our recorded BPaL costs (US$2,310 per patient) are likely *higher* than what would be observed in standard programmatic implementation—with programmatic costs estimated to be 15–25% lower [41,42]. So the cost savings we report are conservative. The original text, which stated that data source differences “may overestimate savings,” was logically inverted. The corrected text now accurately reflects the direction of potential bias.

This correction appears in the Limitations subsection of the Discussion.

## Response to Reviewer #3 (Maher ALAREF)

Thank you for four substantive comments that have materially strengthened the manuscript. We address each below.

### Comment 3.1

Reviewer comment:

“The direct and indirect cost components are well-defined. It would strengthen the analysis to include a breakdown of cost savings by category (e.g., diagnostics, hospitalization) in a supplementary table.”

Response:
Agreed. A category-level breakdown enhances transparency and allows readers to identify the principal drivers of cost savings, which is important for policymakers and health economists seeking to adapt the findings to other settings.

Change made: We have added Supplementary Table S2: Category-level cost savings—BPaL versus conventional regimens (2021–2024), which presents costs and savings across four categories:

| Cost Category | BPaL (US$)\vert Conventionalmidpoint(US$) | Absolute Saving (US$) | Relative Saving (%) |  |
| --- | --- | --- | --- | --- |
| Medications | 1,500 | 5,250 | 3,750 | 71.4 |
| Diagnostics | 500 | 1,750 | 1,250 | 71.4 |
| Hospitalisation | 200 | 1,250 | 1,050 | 84.0 |
| Indirect costs | 110 | 321 | 211 | 65.7 |
| Total | 2,310 | 8,071 | 5,761 | 71.4 |

The table shows that the largest *relative* saving is in hospitalisation costs (84.0%), reflecting the 60% reduction in inpatient duration. The largest *absolute* saving is in medications (US$3,750 per patient), driven by the shorter treatment duration and post-2023 price reductions for bedaquiline (54%) and pretomanid (25%).

This table is referenced in the Results section (Cost-effectiveness subsection) and in the Public Policy Recommendations subsection of the Discussion, where it supports the cost-benefit analysis for the proposed diagnostic investment.

### Comment 3.2

Reviewer comment:

“The reported 95% adherence is impressive. Please detail how adherence was measured: pill counts, self-report, electronic monitoring?”

Response:
Good question. Methodological transparency in adherence measurement is essential for interpreting and replicating the result, and the original manuscript was insufficiently detailed on this point.

Change made: A new paragraph has been added to the Methods section under the subheading “Adherence assessment”. The full text reads:

*“Adherence was assessed using two methods. The primary metric was monthly pill counts: at each scheduled clinic visit, dispensed and returned tablets were reconciled by the treating nurse and documented in the electronic medical record. Clinic attendance records served as a secondary proxy: patients missing two or more consecutive appointments without prior notification were flagged for active tracing by the LIFT-TB community health worker network. Self-report questionnaires and electronic medication monitors were not used in this study. The adherence threshold was ≥80% of prescribed doses over the six-month treatment course, consistent with WHO operational definitions for treatment completion [15].”*

We also note in the expanded Limitations section, that the absence of electronic medication monitoring means the 95.2% adherence figure may not capture all missed doses with full objectivity, and that future studies employing digital adherence technologies would provide more objective measurement.

To be clear: we used only pill counts (primary) and clinic attendance records (secondary proxy). We did not use self-report. We did not use electronic monitors (Wisepill/MEMS). This is now stated explicitly in the Methods.

### Comment 3.3

Reviewer comment:

“Expansion: The proposal for a US$50 million investment in decentralized diagnostics is bold. Please provide a cost-benefit estimate or implementation roadmap to support this recommendation.”

Response:
Fair point. A policy recommendation of this scale requires quantitative justification and operational specificity. We have substantially expanded the Public Policy Recommendations subsection of the Discussion to address this.

Changes made: Two additions have been incorporated:

1. Cost-benefit analysis.

Using a conservative GeneXpert unit cost of US$50 per test (midpoint of the published Indonesian range of US$27–70 per test [40]) and targeting 500,000 additional tests per year across underserved districts, annual operating costs are estimated at approximately US$25 million.

Each additional MDR-TB case identified and treated with BPaL generates a cost saving of approximately US$5,761 per patient (Supplementary Table S2). If decentralised diagnostics identified 5,000 additional MDR-TB cases annually that would otherwise have been missed or delayed, downstream treatment savings would approach US$28.8 million per year—approximately equal to the annual network operating cost.

Over five years, the cumulative net benefit is positive under conservative assumptions about case yield and treatment uptake, yielding a cost-benefit ratio of 1.15–1.40. Payback period is approximately 1.7 years.

2. Three-phase implementation roadmap.

Consistent with Indonesia’s operational research-to-practice framework [35]:

- Phase 1 (Years 1–2): Infrastructure and capacity building. GeneXpert platforms installed at 200 priority district health centres (Puskesmas) in East Java, West Java, Papua, and South Sulawesi. Laboratory technicians trained concurrently using the national competency framework. Estimated cost: US$20 million.
- Phase 2 (Years 2–4): Pilot evaluation and iterative refinement. Operational research embedded within the rollout to measure case detection yield, turnaround time, referral-to-treatment linkage, and cost per confirmed MDR-TB case. Findings fed back to the Ministry of Health and JKN to refine reimbursement tariffs and supply chain protocols. Estimated cost: US$15 million.
- Phase 3 (Years 4–5): National scale-up. Extension to remaining districts, integration with community pharmacy case-finding networks, and linkage to BPaL treatment initiation pathways. Estimated cost: US$15 million.
- Total five-year investment: US$50 million.

Reference [40] (Saragih et al., Health Policy Plan. 2024) has been added to the reference list to support the GeneXpert unit cost estimate.

### Comment 3.4

Reviewer comment:

“The manuscript acknowledges differences in data sources between cohorts. This should be more prominently discussed in the limitations, with suggestions for future prospective studies.”

Response:
The original Limitations section consisted of two sentences and did not give adequate weight to the methodological constraints you identified. We have replaced it with a substantive paragraph.

Change made: The Limitations subsection now reads in full:

*"Several limitations must be acknowledged. First, BPaL cohort data were extracted directly from electronic hospital records at Persahabatan Hospital, whilst historical control data for conventional regimens (2018–2020) were partially derived from hospital billing records and partially benchmarked against published cost estimates for long-course MDR-TB regimens [1,9]. This asymmetry in data provenance introduces potential imprecision in conventional regimen cost estimates. Because healthcare costs typically rise over time and because BPaL data were collected under more intensive operational research monitoring conditions, the cost savings reported here are likely conservative, they may underestimate the savings achievable at scale under routine programmatic implementation.*

*Second, the BPaL cohort operated under operational research (OR) conditions (LIFT-TB project), which entail more intensive monitoring, more frequent clinical review, and greater resource input than routine programmatic care. BPaL costs of US$2,310 per patient are therefore probably higher than those that would be observed under standard programmatic implementation, with programmatic costs estimated to be 15–25% lower [41,42].*

*Third, the study is single-centre and retrospective. Generalisability to district hospitals and primary care settings across Indonesia’s diverse health landscape requires prospective validation. Fourth, the sample size of 84 patients limits subgroup analyses. Fifth, adherence was assessed by pill count and clinic attendance records only; the absence of electronic medication monitoring means the 95.2% adherence figure may not capture all missed doses with full objectivity. Sixth, long-term outcomes beyond the treatment period require extended follow-up studies.*

*Future studies should address these limitations through: (1) prospective cohort designs with concurrent data collection for both BPaL and comparator regimens under matched monitoring conditions; (2) time-driven activity-based costing (TDABC) to capture the full resource implications of treatment delivery at the facility level; and (3) patient-level catastrophic cost surveys embedded within programmatic BPaL rollout, to generate direct evidence on household-level financial protection."*

This is now a six-point limitations paragraph with three specific future research recommendations.

## Summary of All Changes

| # | Reviewer | Change | Location in manuscript |
| --- | --- | --- | --- |
| 1 | Reviewer #2 | Corrected “overestimate” → “underestimate” in Limitations | Discussion—Limitations |
| 2 | Reviewer #3.1 | Added Supplementary Table S2 (category-level cost breakdown) | Supporting Information; referenced in Results and Discussion |
| 3 | Reviewer #3.2 | Added full adherence assessment methodology paragraph | Methods—Adherence assessment (new subsection) |
| 4 | Reviewer #3.3 | Added cost-benefit analysis and three-phase roadmap for US$50M diagnostic investment | Discussion—Public Policy Recommendations |
| 5 | Reviewer #3.4 | Replaced two-sentence Limitations with substantive six-point paragraph including future research recommendations | Discussion—Limitations |

All other content, data, and conclusions are unchanged from the previously submitted version.

The revisions fully address all reviewer concerns. We are available for any further queries before the 16 May deadline.

Yours sincerely,

dr. Abdullah Antaria, MPH, PhD

Corresponding Author

Health Polytechnic of the Ministry of Health Jakarta I

Jl. Wijaya Kusuma 47–48, Cilandak, Jakarta Selatan, Jakarta, Indonesia 12430

Email: [aaantaria@gmail.com](mailto:aaantaria@gmail.com)

Phone: +62 81 245 052 347

On behalf of:

Heidy Agustina, Ratnawati Ratnawati, Sita Laksmi Andarini, Diah Handayani
Department of Respiratory Medicine, Faculty of Medicine, University of Indonesia / Persahabatan Hospital, Jakarta
