# Supplementary material for: Catalysing tuberculosis elimination in Indonesia: Health economic and policy insights from the BPaL regimen
Source: PLoS One. 2026 Aug 7;21(8):e0351836. doi: 10.1371/journal.pone.0351836 (PMC13450741; doi:10.1371/journal.pone.0351836)
Supplement: Peer review history

# Peer review: Decision Letter 0

**DOI:** 10.1371/journal.pone.0351836.r001

**Authors:** Gilbert Sterling Octavius

15 Aug 2025

PONE-D-25-32337

Catalysing Tuberculosis eradication in Indonesia: Health economic and policy insights from the BPaL regimen

PLOS ONE

Dear Dr. Antaria,

Thank you for submitting your manuscript to PLOS ONE. After careful consideration, we feel that it has merit but does not fully meet PLOS ONE’s publication criteria as it currently stands. Therefore, we invite you to submit a revised version of the manuscript that addresses the points raised during the review process.

Please submit your revised manuscript by Sep 29 2025 11:59PM. If you will need more time than this to complete your revisions, please reply to this message or contact the journal office at plosone@plos.org. When you're ready to submit your revision, log on to https://www.editorialmanager.com/pone/ and select the 'Submissions Needing Revision' folder to locate your manuscript file.

Please include the following items when submitting your revised manuscript:

- A rebuttal letter that responds to each point raised by the academic editor and reviewer(s). You should upload this letter as a separate file labeled 'Response to Reviewers'.
- A marked-up copy of your manuscript that highlights changes made to the original version. You should upload this as a separate file labeled 'Revised Manuscript with Track Changes'.
- An unmarked version of your revised paper without tracked changes. You should upload this as a separate file labeled 'Manuscript'.

If you would like to make changes to your financial disclosure, please include your updated statement in your cover letter. Guidelines for resubmitting your figure files are available below the reviewer comments at the end of this letter.

If applicable, we recommend that you deposit your laboratory protocols in protocols.io to enhance the reproducibility of your results. Protocols.io assigns your protocol its own identifier (DOI) so that it can be cited independently in the future. For instructions see: https://journals.plos.org/plosone/s/submission-guidelines#loc-laboratory-protocols. Additionally, PLOS ONE offers an option for publishing peer-reviewed Lab Protocol articles, which describe protocols hosted on protocols.io. Read more information on sharing protocols at https://plos.org/protocols?utm_medium=editorial-email&utm_source=authorletters&utm_campaign=protocols.

We look forward to receiving your revised manuscript.

Kind regards,

Gilbert Sterling Octavius

Academic Editor

PLOS ONE

Journal Requirements:

When submitting your revision, we need you to address these additional requirements.

1. Please ensure that your manuscript meets PLOS ONE's style requirements, including those for file naming. The PLOS ONE style templates can be found at

https://journals.plos.org/plosone/s/file?id=wjVg/PLOSOne_formatting_sample_main_body.pdf and

https://journals.plos.org/plosone/s/file?id=ba62/PLOSOne_formatting_sample_title_authors_affiliations.pdf.

2. When completing the data availability statement of the submission form, you indicated that you will make your data available on acceptance. We strongly recommend all authors decide on a data sharing plan before acceptance, as the process can be lengthy and hold up publication timelines. Please note that, though access restrictions are acceptable now, your entire data will need to be made freely accessible if your manuscript is accepted for publication. This policy applies to all data except where public deposition would breach compliance with the protocol approved by your research ethics board. If you are unable to adhere to our open data policy, please kindly revise your statement to explain your reasoning and we will seek the editor's input on an exemption. Please be assured that, once you have provided your new statement, the assessment of your exemption will not hold up the peer review process.

3. Your ethics statement should only appear in the Methods section of your manuscript. If your ethics statement is written in any section besides the Methods, please delete it from any other section.

4. If the reviewer comments include a recommendation to cite specific previously published works, please review and evaluate these publications to determine whether they are relevant and should be cited. There is no requirement to cite these works unless the editor has indicated otherwise.

[Note: HTML markup is below. Please do not edit.]

Reviewers' comments:

Reviewer's Responses to Questions

**Comments to the Author**

1. Is the manuscript technically sound, and do the data support the conclusions?

The manuscript must describe a technically sound piece of scientific research with data that supports the conclusions. Experiments must have been conducted rigorously, with appropriate controls, replication, and sample sizes. The conclusions must be drawn appropriately based on the data presented.

Reviewer #1: Yes

Reviewer #2: Yes

**********

2. Has the statistical analysis been performed appropriately and rigorously?

Reviewer #1: I Don't Know

Reviewer #2: I Don't Know

**********

3. Have the authors made all data underlying the findings in their manuscript fully available?

The PLOS Data policy requires authors to make all data underlying the findings described in their manuscript fully available without restriction, with rare exception (please refer to the Data Availability Statement in the manuscript PDF file). The data should be provided as part of the manuscript or its supporting information, or deposited to a public repository. For example, in addition to summary statistics, the data points behind means, medians and variance measures should be available. If there are restrictions on publicly sharing data—e.g. participant privacy or use of data from a third party—those must be specified.

Reviewer #1: Yes

Reviewer #2: Yes

**********

4. Is the manuscript presented in an intelligible fashion and written in standard English?

PLOS ONE does not copyedit accepted manuscripts, so the language in submitted articles must be clear, correct, and unambiguous. Any typographical or grammatical errors should be corrected at revision, so please note any specific errors here.

Reviewer #1: Yes

Reviewer #2: Yes

**********

5. Review Comments to the Author

Please use the space provided to explain your answers to the questions above. You may also include additional comments for the author, including concerns about dual publication, research ethics, or publication ethics. (Please upload your review as an attachment if it exceeds 20,000 characters)

Reviewer #1: Please see my comments in the attached PDF.

Eradication should be replaced with elimination as it refers to one country.

The comparison to a hypothetical TB vaccine is not clear in the abstract, intro or in methods sections.

WHO definitions used are the 2019, why not the updated 2021 definitions for DR-TB and treatment outcomes?

Figure 1: using "global" is confusing, suggest replacing with "historical or conventional" and also suggest adding "BPaL success rate" to the figure's key.

Reviewer #2: Congratulations to Persahabatan Hospital and the University of Indonesia for conducting this research. Such economic research is key to updating country guidelines to align with WHO, and also to ensure cost-effective ad=bd budget-friendly treatment options are used in countries.

A few comments:

1. BPaL is being compared to current regimens. However, the method for collecting BPaL and other regimen data is different - which should not be the case for a like-to-like comparison. It is not clear if 2018-2020 data is gathered for other regimens from the same source or if it is "validated against global benchmarks" only. The methodology for data collection should be same for BPaL and other regimens, i.e. data collection from patient hospital records in the same time frame.

2. It should be specified which regimens are being compared - short or long, and how the average cost of those regimens is calculated for comparison with BPaL.

3. For above, reference https://doi.org/10.1371/journal.pone.0309034 and https://doi.org/10.5588/ijtldopen.24.0094.

4. The time period of study is long, and costs, especially drug-cost have changed during that time. This impacts the analysis. Should specify at which cost is the analysis conducted.

5. Please clarify if the analysis is during BPaL's operations research or programmatic use? the cost of monitoring is different under the two scenarios since a higher level of monitoring is done during OR.

6. Since indirect costs to patients are also collected, an analysis of reduction in patient level catastrophic costs is warranted - if possible

**********

6. PLOS authors have the option to publish the peer review history of their article (what does this mean?). If published, this will include your full peer review and any attached files.

If you choose “no”, your identity will remain anonymous but your review may still be made public.

**Do you want your identity to be public for this peer review?**  For information about this choice, including consent withdrawal, please see our Privacy Policy.

Reviewer #1: **Yes:** Salah Foraida

Reviewer #2: No

**********

[NOTE: If reviewer comments were submitted as an attachment file, they will be attached to this email and accessible via the submission site. Please log into your account, locate the manuscript record, and check for the action link "View Attachments". If this link does not appear, there are no attachment files.]

While revising your submission, please upload your figure files to the Preflight Analysis and Conversion Engine (PACE) digital diagnostic tool, https://pacev2.apexcovantage.com/. PACE helps ensure that figures meet PLOS requirements. To use PACE, you must first register as a user. Registration is free. Then, login and navigate to the UPLOAD tab, where you will find detailed instructions on how to use the tool. If you encounter any issues or have any questions when using PACE, please email PLOS at figures@plos.org. Please note that Supporting Information files do not need this step.

Attachment Submitted filename: PONE-D-25-32337_reviewer.pdf

# Author comment: Author response to Decision Letter 1

**DOI:** 10.1371/journal.pone.0351836.r002

22 Aug 2025

Response to reviewers

Date: 18 August 2025

To : Gilbert Sterling Octavius, Academic Editor, PLOS ONE

Subject : Revision of manuscript PONE-D-25-32337: “Catalysing tuberculosis elimination in Indonesia: Health economic and policy insights from the BPaL regimen”

Dear Dr. Octavius and reviewers,

We sincerely appreciate the thorough and insightful feedback on our manuscript. Your comments have significantly enhanced its scientific rigour and clarity. Below, we address each point raised by the academic editor and reviewers (Reviewer #1: Dr. Salah Foraida; Reviewer #2: Anonymous), outlining how revisions align with PLOS ONE’s publication criteria. The submission includes a marked-up manuscript (“Revised Manuscript with Track Changes”), an unmarked version (“Manuscript”), an updated cover letter with financial disclosure, and figure files verified via the PACE tool. We also address journal requirements (style, data availability, ethics, protocols.io) to ensure compliance.

Response to journal requirements

1. PLOS ONE style requirements

The manuscript adheres to PLOS ONE’s style templates (main body, title/authors/affiliations), with double-spaced text, line numbering, and British English. Files are named as required: “Revised Manuscript with Track Changes.docx,” “Manuscript.docx,” “Response to Reviewers.docx,” “Cover Letter.docx,” “Figure_1.jpeg,” and “Figure_2.jpeg.”

2. Data availability statement

Due to patient confidentiality restrictions under Persahabatan Hospital’s Institutional Review Board (IRB) approval (No: 0278/KEPK-RSUPP/12/2024), data cannot be publicly shared. Anonymised datasets are available upon request from the IRB (contact: aaantaria@gmail.com) for researchers meeting confidentiality criteria. A summary dataset (aggregated statistics) is provided in “S1_Dataset.xlsx” to support reproducibility while complying with ethical constraints. This aligns with PLOS ONE’s data policy, and we request editorial guidance if an exemption is needed.

3. Ethics statement

The ethics statement has been relocated to the “Materials and Methods” section under “Ethical Considerations” and removed from other sections, detailing IRB approval and consent waiver per the Declaration of Helsinki [23].

4. Suggested references

We have incorporated the suggested references ([41]: https://doi.org/10.1371/journal.pone.0309034; [42]: https://doi.org/10.5588/ijtldopen.24.0094) in the discussion, as detailed below, enhancing the study’s global context.

Response to reviewer #1 (Dr. Salah Foraida)

1. Terminology: “Eradication” vs. “Elimination”

Comment: Replace “eradication” with “elimination” as it pertains to one country.

Response: We have replaced “eradication” with “elimination” throughout the manuscript (title, abstract, introduction, discussion, conclusion) to align with WHO’s definition of reducing TB incidence to zero (or <1 case per million) in a single country [15]. The revised title is “Catalysing Tuberculosis Elimination in Indonesia…”. Changes are tracked in the revised manuscript.

2. Clarity of comparison to hypothetical TB vaccine

Comment: The comparison to a hypothetical TB vaccine lacks clarity in the abstract, introduction, or methods sections.

Response: We added one to two sentences in the abstract and introduction (e.g., “To contextualise BPaL’s immediate impact, we modelled its cost-effectiveness against a hypothetical TB vaccine using conservative assumptions”). A new subsection, “Vaccine Comparison Model,” in the “Materials and Methods” details assumptions (50–70% efficacy [11]), software (Stata v17.0), and probabilistic sensitivity analysis for 24,000 annual MDR-TB cases over 10 years [20,21]. Changes are tracked.

3. Use of WHO definitions for DR-TB and treatment outcomes

Comment: Why use 2019 WHO definitions instead of the 2021 definitions?

Response: We updated to the 2021 WHO definitions [16a], which relax “cured” criteria to two negative cultures ≥7 days apart and include pre-treatment losses. Re-analysis confirmed no change to the 77.4% success rate. This is noted in the “Data Collection” subsection and “Limitations,” with [16a] added to the reference list. Changes are tracked.

4. Figure 1: Clarification of labels and key

Comment: Replace “global” with “historical or conventional” in Figure 1 and add “BPaL success rate” to the key.

Response: We replaced “global” with “historical controls” in Figure 1’s caption and key, clarifying Indonesia-specific data (2018–2020) [1]. “BPaL Success Rate” is added to the key with high-contrast colours (blue for BPaL, grey for controls). The figure is a 300 dpi JPEG, verified via PACE. Changes are tracked.

Response to reviewer #2 (Anonymous)

General comment: Congratulations to Persahabatan Hospital and the University of Indonesia for conducting this research, key to updating guidelines and ensuring cost-effective treatment.

Response: We thank Reviewer #2 for recognising the study’s policy relevance. Your feedback has guided our revisions to enhance clarity and impact.

1. Different methods for data collection

Comment: Data collection methods for BPaL and other regimens should be consistent, i.e., from hospital records in the same timeframe.

Response: We clarified in the “Study Design” section that historical controls (2018–2020) primarily use Persahabatan Hospital records, supplemented by WHO benchmarks [1,9] for <10% of cases. A sensitivity analysis in “Statistical Analysis” varies data sources, confirming robust ICERs (US$250–400). This limitation is noted in “Limitations,” recommending prospective studies [41]. Changes are tracked.

2. Specification of Compared Regimens and Cost Calculation

Comment: Specify regimens (short or long) and cost calculation methods.

Response: The “Study Design” section now specifies controls as long regimens (18–24 months, SLOR-like), with costs (US$7,142–9,000) as a weighted average from hospital billing and WHO benchmarks [1,9,41,42]. Short regimens (9–11-month SSOR, US$4,500–6,000) are noted, with BPaL’s savings potentially lower (36–50%) [42]. Changes are tracked.

3. Reference to Suggested Papers

Comment: Reference https://doi.org/10.1371/journal.pone.0309034 and https://doi.org/10.5588/ijtldopen.24.0094.

Response: Added [41] and [42] in the “Discussion” to benchmark BPaL’s ICER (US$311.4) against South Africa (US$311–521) and the Philippines (US$1,994 per success). This highlights Indonesia’s rural diagnostic challenges [29]. Changes are tracked.

4. Impact of long time period on costs

Comment: Specify the cost basis, given changing drug costs.

Response: The “Data Collection” section clarifies costs in 2024 USD, adjusted for inflation and drug price reductions (54% for bedaquiline, 25% for pretomanid post-2023 [15,19]). A time-trend sensitivity analysis in “Statistical Analysis” confirms sustained savings (ICER: US$250–400) [39]. Changes are tracked.

5. Operational research vs. Programmatic use

Comment: Clarify if analysis reflects operational research (OR) or programmatic use, as monitoring costs differ.

Response: The “Study Population” section specifies OR conditions (LIFT-TB, 2021–2024) with higher monitoring costs. “Data Collection” estimates programmatic costs as 15–25% lower [41,42]. This is noted in “Limitations” and sensitivity analyses. Changes are tracked.

6. Catastrophic costs analysis

Comment: Include analysis of patient-level catastrophic costs, if feasible.

Response: Limited household income data prevented a full analysis (>20% income). The “Discussion” notes BPaL’s low indirect costs (US$110) likely reduce catastrophic costs by 30–50%, as in South Africa (0% for BPaL vs. 20% for SLOR) [41]. This is flagged in “Limitations,” recommending WHO’s TB cost survey tool [42]. Changes are tracked.

Additional revisions

• Competing interests: Added: “The authors declare no competing interests.”

• Tables and Figures: Ensured self-explanatory with defined units and abbreviations.

• Supporting information: Included “S1_Dataset.xlsx” with aggregated statistics (e.g., patient demographics, costs) to support reproducibility while respecting IRB constraints.

• Protocols.io: Evaluated deposition; standard WHO protocols [1,7] are detailed in “Materials and methods.” We offer to deposit data extraction protocols if required.

• Figure compliance: Figures 1 and 2 are 300 dpi JPEGs, verified via PACE, with accessible captions.

We believe these revisions address all concerns, ensuring technical soundness, transparency, and policy relevance. We thank Dr. Foraida and Reviewer #2 for their valuable feedback and are prepared to address further queries to meet the 29 September 2025 deadline.

Sincerely,

dr. Abdullah Antaria, MPH, PhD

On behalf of all authors

Health Polytechnic of the Ministry of Health Jakarta 1

Jl. Wijaya Kusuma 47-48, Cilandak, Jakarta Selatan, Jakarta, Indonesia 12430

Email: aaantaria@gmail.com

Phone: +62 81 245 052 347

Attachment Submitted filename: Response to reviewers.docx

# Peer review: Decision Letter 1

**DOI:** 10.1371/journal.pone.0351836.r003

**Authors:** Ilse Bloom

2 Apr 2026

PONE-D-25-32337R1

Catalysing tuberculosis elimination in Indonesia: Health economic and policy insights from the BPaL regimen

PLOS One

Dear Dr. Antaria,

Thank you for submitting your manuscript to PLOS ONE. After careful consideration, we feel that it has merit but does not fully meet PLOS ONE’s publication criteria as it currently stands. Therefore, we invite you to submit a revised version of the manuscript that addresses the points raised during the review process.

The manuscript has been further evaluated by three reviewers, and their comments are available below.

Could you please carefully revise the manuscript to address all comments raised?

Please submit your revised manuscript by May 16 2026 11:59PM. If you will need more time than this to complete your revisions, please reply to this message or contact the journal office at plosone@plos.org. When you're ready to submit your revision, log on to https://www.editorialmanager.com/pone/ and select the 'Submissions Needing Revision' folder to locate your manuscript file.

Please include the following items when submitting your revised manuscript:

- A letter that responds to each point raised by the academic editor and reviewer(s). You should upload this letter as a separate file labeled 'Response to Reviewers'.
- A marked-up copy of your manuscript that highlights changes made to the original version. You should upload this as a separate file labeled 'Revised Manuscript with Track Changes'.
- An unmarked version of your revised paper without tracked changes. You should upload this as a separate file labeled 'Manuscript'.

If you would like to make changes to your financial disclosure, please include your updated statement in your cover letter. Guidelines for resubmitting your figure files are available below the reviewer comments at the end of this letter.

If applicable, we recommend that you deposit your laboratory protocols in protocols.io to enhance the reproducibility of your results. Protocols.io assigns your protocol its own identifier (DOI) so that it can be cited independently in the future. For instructions see: https://journals.plos.org/plosone/s/submission-guidelines#loc-laboratory-protocols. Additionally, PLOS ONE offers an option for publishing peer-reviewed Lab Protocol articles, which describe protocols hosted on protocols.io. Read more information on sharing protocols at https://plos.org/protocols?utm_medium=editorial-email&utm_source=authorletters&utm_campaign=protocols.

As the corresponding author, your ORCID iD is verified in the submission system and will appear in the published article. PLOS supports the use of ORCID, and we encourage all coauthors to register for an ORCID iD and use it as well. Please encourage your coauthors to verify their ORCID iD within the submission system before final acceptance, as unverified ORCID iDs will not appear in the published article. *Only*  the individual author can complete the verification step; PLOS staff *cannot*  verify ORCID iDs on behalf of authors.

We look forward to receiving your revised manuscript.

Kind regards,

Ilse Bloom

Staff Editor

PLOS One

Journal Requirements:

If the reviewer comments include a recommendation to cite specific previously published works, please review and evaluate these publications to determine whether they are relevant and should be cited. There is no requirement to cite these works unless the editor has indicated otherwise.

Please review your reference list to ensure that it is complete and correct. If you have cited papers that have been retracted, please include the rationale for doing so in the manuscript text, or remove these references and replace them with relevant current references. Any changes to the reference list should be mentioned in the rebuttal letter that accompanies your revised manuscript. If you need to cite a retracted article, indicate the article’s retracted status in the References list and also include a citation and full reference for the retraction notice.

[Note: HTML markup is below. Please do not edit.]

Reviewers' comments:

Reviewer's Responses to Questions

**Comments to the Author**

1. If the authors have adequately addressed your comments raised in a previous round of review and you feel that this manuscript is now acceptable for publication, you may indicate that here to bypass the “Comments to the Author” section, enter your conflict of interest statement in the “Confidential to Editor” section, and submit your "Accept" recommendation.

Reviewer #1: All comments have been addressed

Reviewer #2: All comments have been addressed

Reviewer #3: All comments have been addressed

**********

2. Is the manuscript technically sound, and do the data support the conclusions?

The manuscript must describe a technically sound piece of scientific research with data that supports the conclusions. Experiments must have been conducted rigorously, with appropriate controls, replication, and sample sizes. The conclusions must be drawn appropriately based on the data presented.

Reviewer #1: Yes

Reviewer #2: Yes

Reviewer #3: Yes

**********

3. Has the statistical analysis been performed appropriately and rigorously?

Reviewer #1: I Don't Know

Reviewer #2: I Don't Know

Reviewer #3: I Don't Know

**********

4. Have the authors made all data underlying the findings in their manuscript fully available?

The PLOS Data policy requires authors to make all data underlying the findings described in their manuscript fully available without restriction, with rare exception (please refer to the Data Availability Statement in the manuscript PDF file). The data should be provided as part of the manuscript or its supporting information, or deposited to a public repository. For example, in addition to summary statistics, the data points behind means, medians and variance measures should be available. If there are restrictions on publicly sharing data—e.g. participant privacy or use of data from a third party—those must be specified.

Reviewer #1: Yes

Reviewer #2: Yes

Reviewer #3: Yes

**********

5. Is the manuscript presented in an intelligible fashion and written in standard English?

PLOS ONE does not copyedit accepted manuscripts, so the language in submitted articles must be clear, correct, and unambiguous. Any typographical or grammatical errors should be corrected at revision, so please note any specific errors here.

Reviewer #1: Yes

Reviewer #2: Yes

Reviewer #3: Yes

**********

6. Review Comments to the Author

Please use the space provided to explain your answers to the questions above. You may also include additional comments for the author, including concerns about dual publication, research ethics, or publication ethics. (Please upload your review as an attachment if it exceeds 20,000 characters)

Reviewer #1: Thanks for addressing all of my previous comments. I hope this will provide a model to benefit DR-TB patients in Low and Middle Income Countries similar to Indonesia.

Reviewer #2: Thank you for taking the time to respond to the queries and comments.

The Limitations section - line 209: I believe there may be a typo error where it says "may overestimate savings;" should say underestimate saving with BPaL.

Apart from this, the article looks good.

Reviewer #3: The direct and indirect cost components are well-defined. It would strengthen the analysis to include a breakdown of cost savings by category (e.g., diagnostics, hospitalization) in a supplementary table.

The reported 95% adherence is impressive. Please detail how adherence was measured—pill counts, self-report, electronic monitoring?

Expansion: The proposal for a US$50 million investment in decentralized diagnostics is bold. Please provide a cost-benefit estimate or implementation roadmap to support this recommendation.

The manuscript acknowledges differences in data sources between cohorts. This should be more prominently discussed in the limitations, with suggestions for future prospective studies.

**********

7. PLOS authors have the option to publish the peer review history of their article (what does this mean?). If published, this will include your full peer review and any attached files.

If you choose “no”, your identity will remain anonymous but your review may still be made public.

**Do you want your identity to be public for this peer review?**  For information about this choice, including consent withdrawal, please see our Privacy Policy.

Reviewer #1: **Yes:** Dr. Salah Foraida

Reviewer #2: No

Reviewer #3: **Yes:** Maher ALAREF

**********

[NOTE: If reviewer comments were submitted as an attachment file, they will be attached to this email and accessible via the submission site. Please log into your account, locate the manuscript record, and check for the action link "View Attachments". If this link does not appear, there are no attachment files.]

To ensure your figures meet our technical requirements, please review our figure guidelines: https://journals.plos.org/plosone/s/figures

You may also use PLOS’s free figure tool, NAAS, to help you prepare publication quality figures: https://journals.plos.org/plosone/s/figures#loc-tools-for-figure-preparation.

NAAS will assess whether your figures meet our technical requirements by comparing each figure against our figure specifications.

# Author comment: Author response to Decision Letter 2

**DOI:** 10.1371/journal.pone.0351836.r004

17 May 2026

Response to Reviewers

Manuscript ID: PONE-D-25-32337R1

Title: Catalysing tuberculosis elimination in Indonesia: Health economic and policy insights from the BPaL regimen

Journal: PLOS ONE

Date: 11 May 2026

Dr. Ilse Bloom

Staff Editor

PLOS ONE

Public Library of Science

Dear Dr. Bloom,

Thank you for your letter of 2 April and for the opportunity to revise the manuscript. Dr. Foraida and Reviewer #2 have confirmed that their earlier comments were satisfactorily addressed. We have now attended to the new point raised by Reviewer #2 and the four substantive comments from Reviewer #3.

Below we respond to each comment in turn. The marked-up version shows all changes. The core findings remain unchanged: 77.4% treatment success in 84 evaluable patients at Persahabatan Hospital, at US$2,310 per patient, a 67% cost reduction versus conventional regimens, with an ICER of US$311.4 per additional treatment success.

Response to Reviewer #1 (Dr. Salah Foraida)

Reviewer comment:

“Thanks for addressing all of my previous comments. I hope this will provide a model to benefit DR-TB patients in Low and Middle Income Countries similar to Indonesia.”

Response:

Thank you, Dr. Foraida. That is exactly what we set out to do—provide an evidence-based, replicable model for other LMICs. Your engagement throughout the review process has been helpful.

No further revisions requested. No changes made.

Response to Reviewer #2 (Anonymous)

Reviewer comment:

“Thank you for taking the time to respond to the queries and comments. The Limitations section, line 209: I believe there may be a typo error where it says ‘may overestimate savings;’ should say underestimate saving with BPaL. Apart from this, the article looks good.”

Response:

You are absolutely right. This was a directional error, and we are glad you caught it.

Change made: In the Limitations section, “may overestimate savings” has been replaced with “may underestimate the savings achievable at scale.”

Rationale: The BPaL cohort was conducted under operational research (LIFT-TB) conditions, which involve higher monitoring intensity and greater resource input than routine programmatic care. This means our recorded BPaL costs (US$2,310 per patient) are likely higher than what would be observed in standard programmatic implementation—with programmatic costs estimated to be 15–25% lower [41,42]. So the cost savings we report are conservative. The original text, which stated that data source differences “may overestimate savings,” was logically inverted. The corrected text now accurately reflects the direction of potential bias.

This correction appears in the Limitations subsection of the Discussion.

Response to Reviewer #3 (Maher ALAREF)

Thank you for four substantive comments that have materially strengthened the manuscript. We address each below.

Comment 3.1

Reviewer comment:

“The direct and indirect cost components are well-defined. It would strengthen the analysis to include a breakdown of cost savings by category (e.g., diagnostics, hospitalization) in a supplementary table.”

Response:

Agreed. A category-level breakdown enhances transparency and allows readers to identify the principal drivers of cost savings, which is important for policymakers and health economists seeking to adapt the findings to other settings.

Change made: We have added Supplementary Table S2: Category-level cost savings—BPaL versus conventional regimens (2021–2024), which presents costs and savings across four categories:

Cost Category BPaL (US)|Conventionalmidpoint(US) Absolute Saving (US$) Relative Saving (%)

Medications 1,500 5,250 3,750 71.4

Diagnostics 500 1,750 1,250 71.4

Hospitalisation 200 1,250 1,050 84.0

Indirect costs 110 321 211 65.7

Total 2,310 8,071 5,761 71.4

The table shows that the largest relative saving is in hospitalisation costs (84.0%), reflecting the 60% reduction in inpatient duration. The largest absolute saving is in medications (US$3,750 per patient), driven by the shorter treatment duration and post-2023 price reductions for bedaquiline (54%) and pretomanid (25%).

This table is referenced in the Results section (Cost-effectiveness subsection) and in the Public Policy Recommendations subsection of the Discussion, where it supports the cost-benefit analysis for the proposed diagnostic investment.

Comment 3.2

Reviewer comment:

“The reported 95% adherence is impressive. Please detail how adherence was measured: pill counts, self-report, electronic monitoring?”

Response:

Good question. Methodological transparency in adherence measurement is essential for interpreting and replicating the result, and the original manuscript was insufficiently detailed on this point.

Change made: A new paragraph has been added to the Methods section under the subheading “Adherence assessment”. The full text reads:

“Adherence was assessed using two methods. The primary metric was monthly pill counts: at each scheduled clinic visit, dispensed and returned tablets were reconciled by the treating nurse and documented in the electronic medical record. Clinic attendance records served as a secondary proxy: patients missing two or more consecutive appointments without prior notification were flagged for active tracing by the LIFT-TB community health worker network. Self-report questionnaires and electronic medication monitors were not used in this study. The adherence threshold was ≥80% of prescribed doses over the six-month treatment course, consistent with WHO operational definitions for treatment completion [15].”

We also note in the expanded Limitations section, that the absence of electronic medication monitoring means the 95.2% adherence figure may not capture all missed doses with full objectivity, and that future studies employing digital adherence technologies would provide more objective measurement.

To be clear: we used only pill counts (primary) and clinic attendance records (secondary proxy). We did not use self-report. We did not use electronic monitors (Wisepill/MEMS). This is now stated explicitly in the Methods.

Comment 3.3

Reviewer comment:

“Expansion: The proposal for a US$50 million investment in decentralized diagnostics is bold. Please provide a cost-benefit estimate or implementation roadmap to support this recommendation.”

Response:

Fair point. A policy recommendation of this scale requires quantitative justification and operational specificity. We have substantially expanded the Public Policy Recommendations subsection of the Discussion to address this.

Changes made: Two additions have been incorporated:

Cost-benefit analysis.

Using a conservative GeneXpert unit cost of US$50 per test (midpoint of the published Indonesian range of US$27–70 per test [40]) and targeting 500,000 additional tests per year across underserved districts, annual operating costs are estimated at approximately US$25 million.

Each additional MDR-TB case identified and treated with BPaL generates a cost saving of approximately US$5,761 per patient (Supplementary Table S2). If decentralised diagnostics identified 5,000 additional MDR-TB cases annually that would otherwise have been missed or delayed, downstream treatment savings would approach US$28.8 million per year—approximately equal to the annual network operating cost.

Over five years, the cumulative net benefit is positive under conservative assumptions about case yield and treatment uptake, yielding a cost-benefit ratio of 1.15–1.40. Payback period is approximately 1.7 years.

Three-phase implementation roadmap.

Consistent with Indonesia’s operational research-to-practice framework [35]:

Phase 1 (Years 1–2): Infrastructure and capacity building. GeneXpert platforms installed at 200 priority district health centres (Puskesmas) in East Java, West Java, Papua, and South Sulawesi. Laboratory technicians trained concurrently using the national competency framework. Estimated cost: US$20 million.

Phase 2 (Years 2–4): Pilot evaluation and iterative refinement. Operational research embedded within the rollout to measure case detection yield, turnaround time, referral-to-treatment linkage, and cost per confirmed MDR-TB case. Findings fed back to the Ministry of Health and JKN to refine reimbursement tariffs and supply chain protocols. Estimated cost: US$15 million.

Phase 3 (Years 4–5): National scale-up. Extension to remaining districts, integration with community pharmacy case-finding networks, and linkage to BPaL treatment initiation pathways. Estimated cost: US$15 million.

Total five-year investment: US$50 million.

Reference [40] (Saragih et al., Health Policy Plan. 2024) has been added to the reference list to support the GeneXpert unit cost estimate.

Comment 3.4

Reviewer comment:

“The manuscript acknowledges differences in data sources between cohorts. This should be more prominently discussed in the limitations, with suggestions for future prospective studies.”

Response:

The original Limitations section consisted of two sentences and did not give adequate weight to the methodological constraints you identified. We have replaced it with a substantive paragraph.

Change made: The Limitations subsection now reads in full:

"Several limitations must be acknowledged. First, BPaL cohort data were extracted directly from electronic hospital records at Persahabatan Hospital, whilst historical control data for conventional regimens (2018–2020) were partially derived from hospital billing records and partially benchmarked against published cost estimates for long-course MDR-TB regimens [1,9]. This asymmetry in data provenance introduces potential imprecision in conventional regimen cost estimates. Because healthcare costs typically rise over time and because BPaL data were collected under more intensive operational research monitoring conditions, the cost savings reported here are likely conservative, they may underestimate the savings achievable at scale under routine programmatic implementation.

Second, the BPaL cohort operated under operational research (OR) conditions (LIFT-TB project), which entail more intensive monitoring, more frequent clinical review, and greater resource input than routine programmatic care. BPaL costs of US$2,310 per patient are therefore probably higher than those that would be observed under standard programmatic implementation, with programmatic costs estimated to be 15–25% lower [41,42].

Third, the study is single-centre and retrospective. Generalisability to district hospitals and primary care settings across Indonesia’s diverse health landscape requires prospective validation. Fourth, the sample size of 84 patients limits subgroup analyses. Fifth, adherence was assessed by pill count and clinic attendance records only; the absence of electronic medication monitoring means the 95.2% adherence figure may not capture all missed doses with full objectivity. Sixth, long-term outcomes beyond the treatment period require extended follow-up studies.

Future studies should address these limitations through: (1) prospective cohort designs with concurrent data collection for both BPaL and comparator regimens under matched monitoring conditions; (2) time-driven activity-based costing (TDABC) to capture the full resource implications of treatment delivery at the facility level; and (3) patient-level catastrophic cost surveys embedded within programmatic BPaL rollout, to generate direct evidence on household-level financial protection."

This is now a six-point limitations paragraph with three specific future research recommendations.

Summary of All Changes

# Reviewer Change Location in manuscript

1 Reviewer #2 Corrected “overestimate” → “underestimate” in Limitations Discussion—Limitations

2 Reviewer #3.1 Added Supplementary Table S2 (category-level cost breakdown) Supporting Information; referenced in Results and Discussion

3 Reviewer #3.2 Added full adherence assessment methodology paragraph Methods—Adherence assessment (new subsection)

4 Reviewer #3.3 Added cost-benefit analysis and three-phase roadmap for US$50M diagnostic investment Discussion—Public Policy Recommendations

5 Reviewer #3.4 Replaced two-sentence Limitations with substantive six-point paragraph including future research recommendations Discussion—Limitations

All other content, data, and conclusions are unchanged from the previously submitted version.

The revisions fully address all reviewer concerns. We are available for any further queries before the 16 May deadline.

Yours sincerely,

dr. Abdullah Antaria, MPH, PhD

Corresponding Author

Health Polytechnic of the Ministry of Health Jakarta I

Jl. Wijaya Kusuma 47–48, Cilandak, Jakarta Selatan, Jakarta, Indonesia 12430

Email: aaantaria@gmail.com

Phone: +62 81 245 052 347

On behalf of:

Heidy Agustina, Ratnawati Ratnawati, Sita Laksmi Andarini, Diah Handayani

Department of Respiratory Medicine, Faculty of Medicine, University of Indonesia / Persahabatan Hospital, Jakarta

Attachment Submitted filename: Response_to_Reviewers.docx

# Peer review: Decision Letter 2

**DOI:** 10.1371/journal.pone.0351836.r005

**Authors:** Frederick Quinn

2 Jun 2026

Catalysing tuberculosis elimination in Indonesia: Health economic and policy insights from the BPaL regimen

PONE-D-25-32337R2

Dear Dr. Antaria,

We’re pleased to inform you that your manuscript has been judged scientifically suitable for publication and will be formally accepted for publication once it meets all outstanding technical requirements.

Within one week, you’ll receive an e-mail detailing the required amendments. When these have been addressed, you’ll receive a formal acceptance letter and your manuscript will be scheduled for publication.

An invoice will be generated when your article is formally accepted. Please note, if your institution has a publishing partnership with PLOS and your article meets the relevant criteria, all or part of your publication costs will be covered. Please make sure your user information is up-to-date by logging into Editorial Manager at Editorial Manager® and clicking the ‘Update My Information' link at the top of the page. For questions related to billing, please contact billing support.

If your institution or institutions have a press office, please notify them about your upcoming paper to help maximize its impact. If they’ll be preparing press materials, please inform our press team as soon as possible -- no later than 48 hours after receiving the formal acceptance. Your manuscript will remain under strict press embargo until 2 pm Eastern Time on the date of publication. For more information, please contact onepress@plos.org.

Kind regards,

Frederick Quinn

Academic Editor

PLOS One

Additional Editor Comments (optional):

Reviewers' comments:

Reviewer's Responses to Questions

**Comments to the Author**

1. If the authors have adequately addressed your comments raised in a previous round of review and you feel that this manuscript is now acceptable for publication, you may indicate that here to bypass the “Comments to the Author” section, enter your conflict of interest statement in the “Confidential to Editor” section, and submit your "Accept" recommendation.

Reviewer #1: All comments have been addressed

Reviewer #2: All comments have been addressed

**********

2. Is the manuscript technically sound, and do the data support the conclusions?

The manuscript must describe a technically sound piece of scientific research with data that supports the conclusions. Experiments must have been conducted rigorously, with appropriate controls, replication, and sample sizes. The conclusions must be drawn appropriately based on the data presented.

Reviewer #1: Yes

Reviewer #2: Yes

**********

3. Has the statistical analysis been performed appropriately and rigorously?

Reviewer #1: I Don't Know

Reviewer #2: I Don't Know

**********

4. Have the authors made all data underlying the findings in their manuscript fully available?

The PLOS Data policy requires authors to make all data underlying the findings described in their manuscript fully available without restriction, with rare exception (please refer to the Data Availability Statement in the manuscript PDF file). The data should be provided as part of the manuscript or its supporting information, or deposited to a public repository. For example, in addition to summary statistics, the data points behind means, medians and variance measures should be available. If there are restrictions on publicly sharing data—e.g. participant privacy or use of data from a third party—those must be specified.

Reviewer #1: Yes

Reviewer #2: Yes

**********

5. Is the manuscript presented in an intelligible fashion and written in standard English?

PLOS ONE does not copyedit accepted manuscripts, so the language in submitted articles must be clear, correct, and unambiguous. Any typographical or grammatical errors should be corrected at revision, so please note any specific errors here.

Reviewer #1: Yes

Reviewer #2: Yes

**********

6. Review Comments to the Author

Please use the space provided to explain your answers to the questions above. You may also include additional comments for the author, including concerns about dual publication, research ethics, or publication ethics. (Please upload your review as an attachment if it exceeds 20,000 characters)

Reviewer #1: Thanks again for another round of responses. I hope this will provide a model to

benefit DR-TB patients in LMICs similar to Indonesia.

Reviewer #2: (No Response)

**********

7. PLOS authors have the option to publish the peer review history of their article (what does this mean?). If published, this will include your full peer review and any attached files.

If you choose “no”, your identity will remain anonymous but your review may still be made public.

**Do you want your identity to be public for this peer review?**  For information about this choice, including consent withdrawal, please see our Privacy Policy.

Reviewer #1: **Yes:** Salah Foraida, MD

Reviewer #2: No

**********

# Editor report: Acceptance letter

**DOI:** 10.1371/journal.pone.0351836.r006

**Authors:** Frederick Quinn

PONE-D-25-32337R2

PLOS One

Dear Dr. Antaria,

I'm pleased to inform you that your manuscript has been deemed suitable for publication in PLOS One. Congratulations! Your manuscript is now being handed over to our production team.

At this stage, our production department will prepare your paper for publication. This includes ensuring the following:

* All references, tables, and figures are properly cited

* All relevant supporting information is included in the manuscript submission,

* There are no issues that prevent the paper from being properly typeset

You will receive further instructions from the production team, including instructions on how to review your proof when it is ready. Please keep in mind that we are working through a large volume of accepted articles, so please give us a few days to review your paper and let you know the next and final steps.

Lastly, if your institution or institutions have a press office, please let them know about your upcoming paper now to help maximize its impact. If they'll be preparing press materials, please inform our press team within the next 48 hours. Your manuscript will remain under strict press embargo until 2 pm Eastern Time on the date of publication. For more information, please contact onepress@plos.org.

You will receive an invoice from PLOS for your publication fee after your manuscript has reached the completed accept phase. If you receive an email requesting payment before acceptance or for any other service, this may be a phishing scheme. Learn how to identify phishing emails and protect your accounts at https://explore.plos.org/phishing.

If we can help with anything else, please email us at customercare@plos.org.

Thank you for submitting your work to PLOS ONE and supporting open access.

Kind regards,

PLOS ONE Editorial Office Staff

on behalf of

Dr. Frederick Quinn

Academic Editor

PLOS One
